# Enterotoxigenic *Escherichia coli*–induced intestinal epithelial necroptosis drives lamina propria immune cell pyroptosis and mucosal injury in piglets

**DOI:** 10.3389/fimmu.2026.1778258

**Published:** 2026-03-19

**Authors:** Xiaoyu Wu, Yujiao Liu, Shaofeng Wu, Hongkui Wei, Jian Peng

**Affiliations:** 1 Department of Animal Nutrition and Feed Science, College of Animal Science and Technology, Huazhong Agricultural University, Wuhan, China; 2 The Cooperative Innovation Center for Sustainable Pig Production, Wuhan, China

**Keywords:** Escherichia coli, intestinal inflammation, necroptosis, piglet, pyroptosis

## Abstract

Necroptosis is an inflammatory programmed cell death pathway linked to diverse physiological and pathological disorders, yet its role in Enterotoxigenic *Escherichia coli* (ETEC)−induced intestinal inflammation and mucosal injury remains poorly understood. This study aimed to elucidate the contribution of intestinal epithelial cell necroptosis to the development of intestinal inflammation and injury induced by ETEC infection in piglets. Following ETEC challenge in piglets, key proteins involved in necroptosis, including phosphorylated receptor-interacting protein kinase 3 (p-RIP3) and high-mobility group box 1 (HMGB1), were upregulated in jejunal crypt epithelial cells, which are primarily composed of Paneth cells and stem cells, in a time-dependent manner. In addition, ETEC challenge triggered time−dependent pyroptosis in jejunal lamina propria lymphocytes, a population that includes macrophages, as demonstrated by elevated levels of NLRP3, Caspase−1, GSDMD−N, and Cleaved -IL−1β (p17) proteins in lamina propria lymphocytes. Necroptosis of jejunal crypt epithelial cells occurred prior to pyroptosis of lamina propria lymphocytes, indicating that epithelial cell necroptosis may contribute to the induction of pyroptosis in lamina propria lymphocytes. ETEC challenge induced progressive TNF−α and IL−1β upregulation in plasma, jejunal crypt epithelial cells, and lamina propria lymphocytes of piglets. These changes coincided with intestinal injury and barrier loss, which were indicated by increased plasma i−FABP and decreased jejunal ZO−1 and Occludin. Notably, Nec−1 pretreatment mitigates ETEC−induced intestinal inflammation and tissue damage in piglets by inhibition of crypt epithelial cells necroptosis and the ensuing pyroptosis of lymphocytes. These results indicate that targeting upstream epithelial-cell necroptosis is an important strategy to attenuate inflammation and preserve barrier integrity.

## 1 Introduction

Enterotoxigenic *Escherichia coli* (ETEC) infection is a major cause of diarrhoea in neonatal and post-weaning piglets and results in dehydration, anorexia, weight loss, and sudden death (1). During outbreaks of ETEC, pig mortality increased from 2% to 7%, resulting in significant economic losses (2). Pathogenic infection often results in inflammatory programmed cell death, including pyroptosis, necroptosis, and PANoptosis, thus aggravating inflammation and injury (3, 4). Necroptosis induces by pathogenic infection leads to cell lysis, which results in the release of intracellular damage-associated molecular patterns (DAMPs) and pathogen-associated molecular patterns (PAMPs) (4). The necroptosis signaling pathway is activated by stimulation of tumor necrosis factor receptor 1 (TNFR1) or Toll-like receptor (TLR) (5, 6). Upon activation, receptor-interacting protein 1 (RIP1) recruits RIP3 to form the necrosome, leading to the phosphorylation of mixed-lineage kinase domain-like protein (MLKL) (7). Phosphorylated MLKL is translocated to the plasma membrane, leading to membrane rupture and cell lysis (8). Previous studies have reported that IPEC-1 cells treated with ETEC can induce the upregulation of necroptosis-related biomarkers, such as phosphorylated RIP1, p-RIP3, and p-MLKL (9, 10). Despite extensive studies on ETEC infection, direct *in vivo* evidence of necroptosis in small intestinal epithelial cells of piglets is still lacking, and the extent to which necroptosis contributes to the inflammatory intestinal pathology induced by ETEC in piglets remains unresolved.

Intracellular DAMPs and d PAMPs are capable of activating the cytosolic pattern recognition receptor NOD-like receptor family pyrin domain-containing 3 (NLRP3). Upon activation, NLRP3 recruits apoptosis-associated speck-like protein (ASC) and pro-caspase-1 to form the NLRP3 inflammasome, which subsequently results in the activation of caspase-1. Upon activation, caspase-1 promotes the maturation of IL-1β and IL-18, and cleaves GSDMD to produce the pore-forming GSDMD-N fragment, thereby enabling the release of large quantities of inflammatory cytokines and driving pyroptotic cell death (11, 12). Inflammatory mediators and DAMPs released during pyroptotic cell death further compromise the integrity of the intestinal epithelial barrier (13–15). It is noteworthy that DAMPs generated by necroptotic intestinal epithelial cells facilitate the induction of pyroptosis via TLR-mediated signaling in macrophages (16). A recent study revealed that ETEC infection induces NLRP3 inflammasome–mediated pyroptosis in peripheral blood mononuclear cells of weaned piglets (17). In addition, evidence has demonstrated that ETEC infection enhances the expression of pyroptosis-associated proteins in the jejunal mucosa of weaned piglets (18). Notably, no studies to date have demonstrated that ETEC infection in weaned piglets induces pyroptosis in lamina propria macrophages, nor have any investigations established a link between ETEC-induced necroptosis of intestinal epithelial cells and pyroptosis of lamina propria macrophages. Building on prior literature, we propose that ETEC-induced epithelial necroptosis drives pyroptosis in lamina propria macrophages, which is central to the development of intestinal inflammation and barrier injury.

In the present study, we first demonstrated that ETEC infection induces necroptosis in intestinal epithelial cells of piglets and triggers pyroptosis in lamina propria macrophages. We employed pretreatment with the necroptosis inhibitor necrostatin−1 (Nec−1) to investigate the role of epithelial cell necroptosis in the regulation of pyroptosis in lamina propria lymphocytes. This study provides insights into the pathogenic mechanisms of ETEC and may offer novel strategies to mitigate ETEC-induced intestinal inflammation and injury.

## 2 Material and methods

### 2.1 Bacterial strain and culture conditions

AThe specific ETEC strain used for the challenge experiments is the classic reference strain CVCC196 (original designation C83902/G205), obtained from the China Veterinary Culture Collection Center (CVCC; http://cvcc.ivdc.org.cn/). Originating from the United Kingdom, this wild-type strain was initially isolated from the intestinal mucosa of a piglet. Biologically, it is a highly pathogenic reference strain characterized by the serotype O8: K87, K88ac. To confirm the genetic stability and virulence profile of the strain prior to the organoid infection assay, we performed PCR analysis. As shown in **Supplementary Figure 3B**, the genetic verification confirmed that this strain robustly carries genes for the O8 somatic antigen, the F4ac (formerly K88ac) fimbriae responsible for specific epithelial adhesion, and the enterotoxins LT (heat-labile) and STb (heat-stable). The bacteria were routinely cultured in LB broth at 37 °C. The ETEC CVCC196 strain used in this study is a well-characterized porcine pathogen. It has been successfully employed by Xia et al. (19) to establish neonatal piglet diarrhea models. Furthermore, Osman et al. (20) utilized this specific strain to evaluate the antibacterial efficacy of robust goat-derived Enterococcus species. Additionally, *in vitro* studies by Yang et al. have demonstrated that ETEC CVCC196 possesses significant hemolytic activity, further confirming its virulence potential.

### 2.2 Animals and experimental design

All experiments were approved by the Animal Experimental Ethical Inspection Committee of the Laboratory Animal Centre, Huazhong Agricultural University (HZAUSW-2025-0042). Forty-two weanling pigs (male, Duroc × Large White × Landrace, 21 d, average body weight 6.1 ± 0.2 kg BW) were purchased from Macheng Xiongfeng Ecological Animal Husbandry Co., Ltd. (Hubei, China). Piglets were housed individually in metabolism crates in an environmentally controlled facility. Throughout the experimental period, piglets had ad libitum access to a conventional milk replacer and water. On day 2 after arrival, piglets were orally administered streptomycin sulfate (20 mg/kg BW) to disrupt the gut microbiota and thereby enhance susceptibility to ETEC colonization. ETEC was grown overnight in Luria-Bertani (LB) broth media at 37 °C under continuous shaking (200 rpm). An 18-hour ETEC culture was introduced into LB at a concentration of 0.1% (v/v), followed by 5 h of growth at 37 °C with constant shaking. Bacterial enumeration was performed through OD600 measurements using a spectrophotometer, according to the established conversion: OD600 of 1.0 = 4.8 × 10^8^ cells/mL. ETEC used for piglet challenge were obtained by centrifuging bacterial cultures at 4000 × g for 10 minutes, washing the pellets three times with sterile PBS, and resuspending them in sterile LB broth to a final concentration of 2 × 10^9^ CFU/mL. At 24 h after streptomycin sulfate gavage, piglets were orally administered an LB solution containing 5% sodium bicarbonate (5 mL/kg BW), followed by an ETEC challenge at a dose of 1 × 10^10^ CFU/kg BW. Nec−1 pretreatment in piglets was performed according to the protocol described by Liu et al (21). Piglets received an intraperitoneal injection of Nec-1 (1.0 mg/kg BW) or an equal volume of physiological saline containing 2% DMSO, administered 30 minutes prior to the ETEC challenge. Piglets in the ETEC challenge group were euthanized and sampled at 0, 2, 4, and 8 h after ETEC challenge. For the ETEC−challenged plus Nec−1−treated group, euthanasia and sampling were performed at 2, 4, and 8 h post−ETEC challenge.

### 2.3 Blood sample and tissue collections

Blood samples (8 mL) were obtained from the anterior vena cava before euthanasia, collected in sodium heparin anticoagulant tubes, centrifuged for plasma separation, and stored at −80 °C for further analysis. Following blood sampling, piglets were humanely euthanized by trained personnel using intravenous injection of pentobarbital sodium (80 mg/kg BW) via the ear marginal vein. Jejunal segments measuring 3 cm, 10 cm, and 5 cm in length were collected from a site approximately 1.5 m proximal to the ileocecal junction. The 3 cm intestinal segment was rinsed thoroughly with cold PBS, fixed in 4% paraformaldehyde, and used for subsequent morphological analysis. After being opened longitudinally, the 10 cm intestinal segment was rinsed with PBS to remove luminal contents, and residual PBS on the mucosal surface was blotted dry with absorbent paper. Intestinal mucosal samples were scraped using microscope slides, collected into 5 mL cryogenic tubes, homogenized with a sterile cotton swab, snap-frozen in liquid nitrogen, and stored at −80 °C until further analysis. The 5 cm intestinal segment was used for the isolation of crypt epithelial cells and lamina propria lymphocytes.

### 2.4 Isolation of crypt epithelial cells and lamina propria lymphocytes

After being opened longitudinally, the 5 cm intestinal segment was rinsed with cold Ca²^+^/Mg²^+^−free DPBS, and the external muscularis and serosal layers were carefully removed by dissection. The tissue was minced into approximately 1 cm fragments using ophthalmic scissors. The tissue fragments were incubated in an epithelial dissociation solution consisting of 20 mL HBSS, 1% penicillin–streptomycin, 1% HEPES, 5 mM EDTA, and 0.94 mol/L dithiothreitol (DTT), with continuous shaking at 37 °C and 200 rpm for 40 min. Subsequently, the digestion mixture containing the tissue fragments was transferred from the 50 mL conical flask to a 50 mL centrifuge tube and gently shaken horizontally for 2 min to remove the apical epithelial cells. The tissue fragments collected after filtration were transferred into a conical flask containing 20 mL of epithelial digestion solution and subjected to an additional 40 min of digestion under the same conditions as above. The digestion was performed in three successive rounds. After the final round, the tube was vigorously shaken by hand for 2 min, and the resulting suspension was filtered through a 100-µm cell strainer to obtain a fraction enriched in crypt epithelial cells. Tissue fragments were first gently shaken for approximately 2 min in 20 mL Ca²^+^/Mg²^+^−free HBSS, then transferred to 20 mL RPMI-1640 medium supplemented with 1% penicillin–streptomycin, 2 mM HEPES, 2 mg DNase I, and 2% fetal bovine serum (FBS), and incubated with shaking (37 °C, 200 rpm) for 30 min to restore ionic homeostasis. After gentle shaking, the fragments were collected and incubated in 10 mL RPMI-1640 medium supplemented with 1% penicillin–streptomycin, 2 mM HEPES, 10 mg DNase I, 50 mg collagenase VIII, and 1% heat-inactivated FBS at 37 °C with shaking at 200 rpm for 60 min. The collected digestive fluid was supplemented with culture medium (10 mL RPMI-1640 containing 5% FBS) and centrifuged at 400 × g for 10 min at 4 °C. The resulting pellet was resuspended in 40% Percoll solution (GE Healthcare), carefully layered onto 80% Percoll solution, and centrifuged at 800 × g for 20 min at 4 °C. Lymphocytes were isolated from the interface between the 40% and 80% Percoll layers, washed three times in buffer, cryopreserved in liquid nitrogen, and stored at −80 °C until further use.

### 2.5 Isolation and verification of mucosal-adhered ETEC

To assess the colonization of the challenge strain on the intestinal epithelia, jejunal mucosal samples were collected at 0, 4, and 8 h post-infection. A 1-cm segment of the jejunum was excised approximately 1.5 m proximal to the ileocecal junction. Each segment was opened longitudinally with sterile scissors and rinsed repeatedly with sterile PBS to remove intestinal contents and bulk mucus. To ensure the removal of non-adherent (luminal) bacteria, the intestinal segments were transferred to 50-mL sterile centrifuge tubes containing 30 mL of sterile PBS. The tubes were shaken vigorously by hand for 3 minutes within a laminar flow hood. This washing process was repeated at least five times using fresh sterile PBS until the supernatant remained colorless and clear. Following the final wash, the jejunal tissue was placed on a sterile glass plate, and the mucosal layer was gently scraped off using a sterile microscope slide. For bacterial isolation, a portion of the collected mucosal tissue was transferred into a 2-mL sterile microcentrifuge tube containing 1 mL of sterile PBS and a sterile grinding bead. The samples were fully homogenized and then streaked onto MacConkey agar plates. After overnight incubation, representative single colonies were randomly selected and cultured in LB broth at 37 °C. Bacterial cells were harvested by centrifugation (4000 × g, 10 min), and genomic DNA was released using the boiling lysis method. Briefly, the cell pellets were resuspended in sterile water, boiled at 100 °C for 10 min, and centrifuged; the resulting supernatant was used as the DNA template. The genotypic identity of the isolates—including serotype (O8), fimbriae (F4ac), and enterotoxins (LT and STb)—was confirmed by PCR using the specific primers listed in **Supplementary Table 1**.

### 2.6 Intestinal morphology

Jejunal segments were fixed in 4% paraformaldehyde for 24 h at room temperature, dehydrated through a graded ethanol series, cleared in xylene, and embedded in paraffin. The paraffin blocks were sectioned at 5 µm thickness and stained with hematoxylin and eosin (H&E). Villus height and crypt depth were measured from five well-oriented crypt-villus units per sample. These measurements were averaged to provide a single representative value per biological replicate, and the Villus height-to-crypt depth (V/C) ratio was subsequently calculated.

### 2.7 Enzyme-linked immunosorbent assay

The levels of i-FABP, IL−1β, and TNF−α in plasma, as well as high-mobility group box 1 (HMGB1), IL−1β, and TNF−α in jejunal crypt epithelial cells, were quantified using porcine−specific ELISA kits (Jiangsu Meimian Industrial Co., Ltd.) according to the manufacturer’s instructions. Results for the crypt epithelial cell supernatant were expressed as pg per mg of protein.

### 2.8 Western blot analysis of protein expression

Protein lysates were prepared from jejunal mucosal tissue and lamina propria lymphocytes using Radio Immunoprecipitation Assay (RIPA) lysis buffer (P0013B, Beyotime) supplemented with a protease inhibitor cocktail. The protein concentration of the lysates was determined using a BCA Protein Assay Kit (P0009, Beyotime), and then adjusted to the same level with RIPA lysis buffer containing a protease inhibitor cocktail. Protein lysates were mixed with 5× SDS−PAGE loading buffer, heated at 95 °C for 10 min to denature proteins, and then used for subsequent analyses. Denatured proteins were separated by SDS-PAGE and subsequently transferred to nitrocellulose (NC) membranes (HATF85R, Immobilon). The NC membrane was blocked with rapid blocking solution (PS108P, Epizyme) for 10 min and then incubated with the primary and secondary antibodies. The primary antibodies: β-Actin (AC026, Abclonal, 1:80000), ZO-1 (21773-1-AP, Proteintech, 1:5000), Occludin (80545-1-RR, Proteintech, 1:5000), Caspase 1 (22915-1-AP, Proteintech, 1:2000), IL-1β (16806-1-AP, Proteintech, 1:2000), GSDMD (A24476, Abclonal, 1:1000), NLRP3 (A5652, Abclonal, 1:1000). Second antibodies: HRP-conjugated Goat anti-Rabbit IgG (H+L) (AS014, Abclonal, 1:5000). Protein band intensities were quantified by densitometric analysis using ImageJ software (NIH, Bethesda, MD, USA). The integrated density of each target band was measured and normalized to its respective internal loading control to determine relative protein expression levels.

### 2.9 Immunofluorescence staining

Jejunal tissue from piglets was fixed in 4% paraformaldehyde and subsequently embedded in paraffin. Paraffin−embedded sections were subjected to immunofluorescence staining, in which primary antibodies specific to the target antigens were incubated with the sections, followed by incubation with appropriate fluorophore−conjugated secondary antibodies. Fluorescence signals were visualized using a fluorescence microscope (U−LH100HG, Olympus Corporation, Tokyo, Japan). The primary antibodies for immunofluorescence: anti-ZO-1 (21773-1-AP, Proteintech, 1:100), anti-p-RIP3 (91702, CST, 1:200), anti-HMGB1 (10829-1-AP, Proteintech, 1:200), anti-CD68 (GB113109, Servicebio, 1:100), anti-Cleaved GSDMD (N Terminal) (A22523, Abclonal, 1:100). Secondary antibodies: Alexa Fluor^®^ 488 AffiniPure Goat Anti-Rabbit IgG (111–545-003, Jackson ImmunoResearch), Cy™3 AffiniPure Goat Anti-Rabbit IgG (111–165-003, Jackson ImmunoResearch) and Goat anti-Rabbit IgG (H+L) Cross-Adsorbed Secondary Antibody, Alexa Fluor^®^ 555, (A-21428, Thermo Fisher Scientific). One representative image was obtained from each piglet in every experimental group. The expression of all target proteins was quantified by measuring the mean fluorescence intensity (MFI) using ImageJ software. For ZO-1 and p-RIP3, three biological replicates per group were analyzed: eight intact villi were randomly selected per section for ZO-1 (total 24 villi/group), and twelve well-defined crypts were identified for p-RIP3 (total 36 crypts/group). In contrast, for HMGB1, CD68, and GSDMD-N, one representative biological sample per group was processed, from which 15 well-defined crypts (for HMGB1) or 15 intact villi (for CD68 and GSDMD-N) were randomly selected. To capture the full spectrum of signaling intensity and distribution, each measured crypt or villus in this second set was treated as an independent observation unit, with all 15 individual measurements per group included in the final analysis.

### 2.10 RNA extraction and quantitative real-time PCR

Total RNA was isolated from jejunal mucosa, jejunal crypt epithelial cells, and lamina propria lymphocytes using TRIzol reagent (ABclonal, China; Catalog No. RK30129). cDNA was synthesized from 1 μg of mRNA using the ABScript Neo RT Master Mix for qPCR with gDNA Remover (ABclonal, China, Catalog RK20433). Relative gene expression quantification was performed using the ChamQ SYBR qPCR Master Mix (Without ROX) (Vazyme, China, Catalog Q321-02), with subsequent analysis conducted through the “2^-ΔΔCt^” calculation method. **Supplementary Table 1** contains the complete list of primer sequences used in the current study (22, 23).

### 2.11 Statistical analysis

Data were analyzed using SPSS (version 23.0, SPSS Inc., Chicago, IL) and presented as the mean ± SEM. Data normality was assessed using the Shapiro-Wilk test. All statistical analyses conducted in this study were unpaired. For the ETEC infection time-course study, each post-infection time point was compared independently to the 0 h control group; no comparisons were made between different time points post-infection. For the intervention study, comparisons were restricted to the ETEC group and the ETEC+Nec-1 group at the same corresponding time points. Because all analyses involved strictly pre-planned, independent pairwise comparisons, no multiple-comparison corrections were applied. For data following a normal distribution, an unpaired two-tailed Student’s t-test was performed. When variances were unequal, Welch’s correction was applied. For data that did not follow a normal distribution, the unpaired non-parametric Mann–Whitney U test was used. For comparisons with a small sample size (n=3 per group), Welch’s t-test (unpaired t-test with Welch’s correction) was employed to determine statistical significance. Statistical significance was established when the *p*-value fell below 0.05.

## 3 Results

### 3.1 Necroptosis in piglet crypt epithelial cells occurs earlier than pyroptosis in lamina propria lymphocytes following ETEC infection

**Figure 1:**
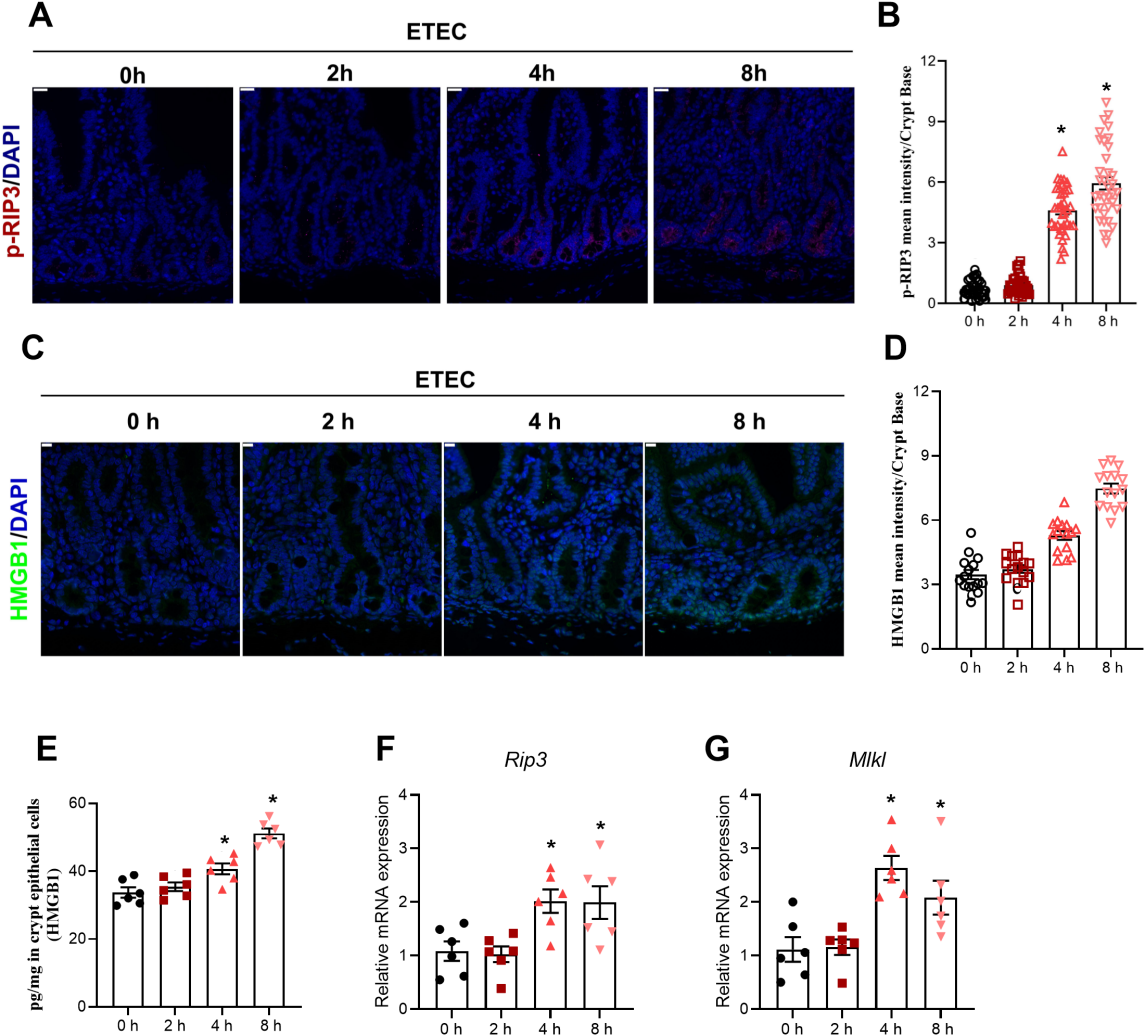
ETEC infection dynamically induces necroptosis in intestinal cells of piglets. **(A)** p-RIP3 staining of jejunum in the indicated groups. Scale bar: 20 μm (top-left corner). **(B)** MFI of p−RIP3 in epithelial cells of the jejunal crypt region. MFI quantification of p-RIP3 at the crypt base (n=3 piglets per group; see “Materials and Methods” for details on the quantification method). **(C)** HMGB1 staining of jejunum in the indicated groups. Scale bar: 10 μm (top-left corner). **(D)** MFI of HMGB1 at the crypt base. To capture the distribution of protein expression, 15 well-defined crypts per group were randomly selected from a single biological sample (n=1 piglet per group) and analyzed as independent observation units. For specific details on the sampling and quantification procedure, see ‘Materials and Methods’. Results are shown as observed values from a single biological replicate, and no statistical tests were applied. **(E)** HMGB1 protein levels (pg/mg) in crypt epithelial cells measured by ELISA (n=6 piglets per group). **(F, G)** mRNA expression levels of *Rip3* and *MLKL* in jejunal crypt epithelial cells (n=6 piglets per group). For the data presented in this figure, a total of 24 piglets were used across the four experimental groups (0 h, 2 h, 4 h, and 8 h). Each time point was compared with the 0 h control after assessing data normality. For normally distributed data, an unpaired two-tailed Student’s t-test was used; Welch’s correction was applied when variances were unequal. For data that did not follow a normal distribution, the Mann–Whitney U test was performed. Data are presented as mean ± SEM. ^*^P < 0.05 vs. the 0 h group.

In this section, a total of 24 piglets were all subjected to ETEC challenge and allocated into four groups based on sampling time (0, 2, 4, and 8 h post-infection), conducted as one independent *in vivo* experiment. The specific number of samples (n) used for each individual assay is detailed in the corresponding figure legends. To investigate the temporal effects of ETEC challenge on intestinal cell death and inflammatory signaling, we dynamically monitored necroptosis markers and HMGB1 expression. Immunofluorescence staining revealed a progressive increase in p-RIP3 levels within the crypt base, with significant elevations observed starting at 4 h and persisting through 8 h post-infection (**Figures 1A, B**). Consistent with the activation of necroptosis, the mRNA levels of key necroptotic executioners, *Rip3* and *Mlkl*, were significantly upregulated at 4 h and 8 h compared to the 0 h control in jejunal crypt epithelial cells (**Figures 1F, G**). Simultaneously, the expression of HMGB1 showed a similar time-dependent increase. The fluorescence intensity of HMGB1 in the crypt base was increased at 4 h and 8 h post-infection (**Figures 1C, D**). Furthermore, quantification of HMGB1 protein levels in isolated crypt epithelial cells via ELISA validated this trend, showing a significant increase of HMGB1 as the infection progressed (**Figure 1E**). In the present study, these findings indicate that ETEC challenge in piglets induced necroptosis in jejunal crypt epithelial cells at 4 h post−infection.

In the present study, ETEC infection was found to primarily induce necroptosis in jejunal crypt epithelial cells of piglets, which mainly consist of stem cells and Paneth cells. Based on this finding, we analyzed the time-course changes in expression levels of the Paneth cell marker Lyz and the stem cell markers Lgr5 and Ascl2 following ETEC challenge. The expression level of *lyz* mRNA was significantly increased at 2–8 h post-infection (**Supplementary Figure 1A**). Compared to the 0 h control group, ETEC challenge decreased *Lgr5* mRNA levels at 4–8 h (**Supplementary Figure 1B**) and *Ascl2* mRNA levels at 8 h (**Supplementary Figure 1C**).

**Figure 2:**
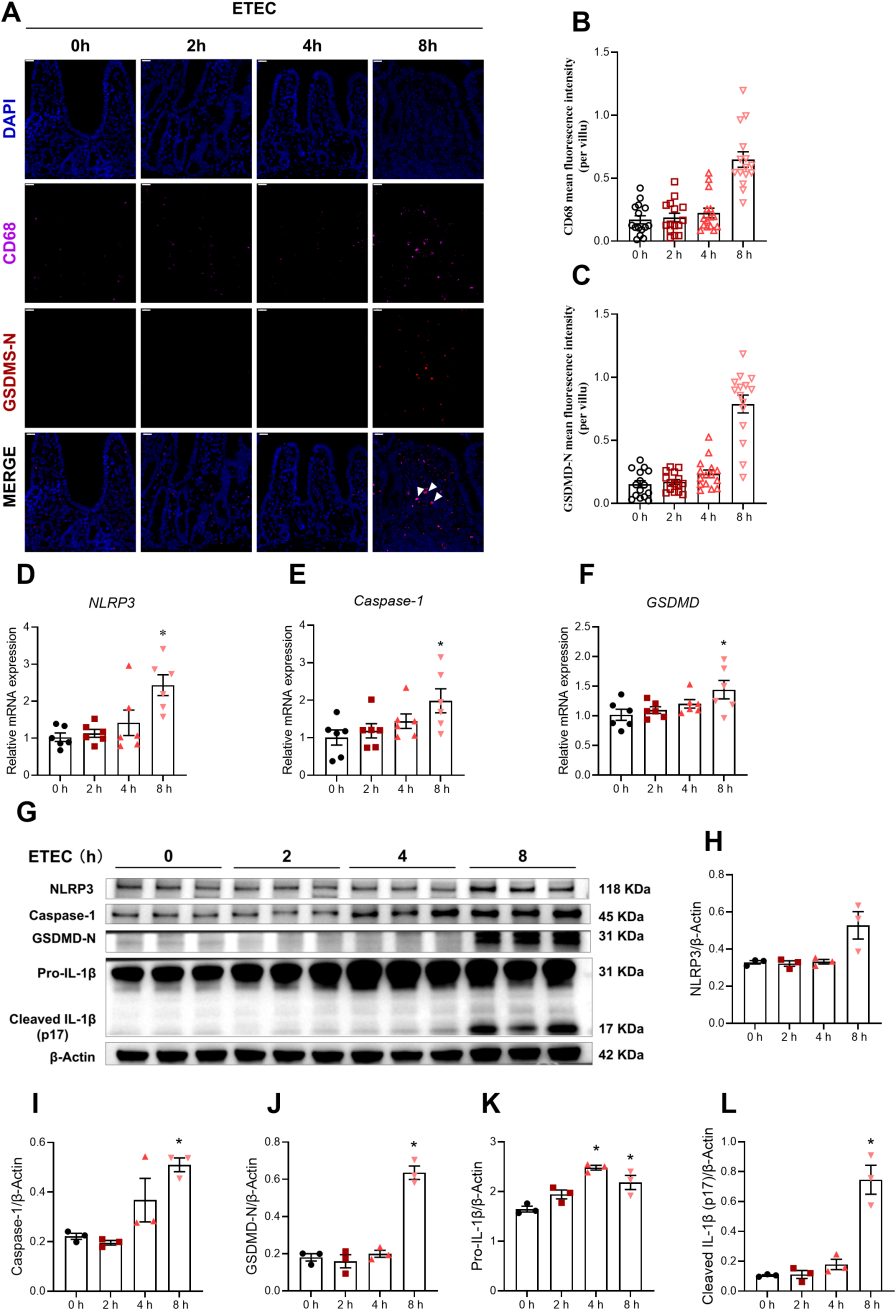
ETEC infection dynamically induces pyroptosis in jejunal lamina propria lymphocytes of piglets. **(A)** CD68 and GSDMD-N staining of jejunum in the indicated groups. White arrows indicate the colocalization of GSDMD-N and CD68. Scale bar: 20 μm (top-left corner). **(B, C)** MFI for CD68 and GSDMD-N. To capture the distribution of protein expression, 15 well-defined villi per group were randomly selected from a single biological sample (n=1 piglet per group) and analyzed as independent observation units. For specific details on the sampling and quantification procedure, see ‘Materials and Methods’. Results are shown as observed values from a single biological replicate, and no statistical tests were applied. **(D–F)** mRNA expression levels of Caspase-1, NLRP3 and GSDMD in jejunal lamina propria lymphocytes (n=6 piglets per group). **(G)** Western blot analysis of NLRP3, Caspase-1, GSDMD-N, Pro-IL-1β, and Cleaved IL-1β (p17) in the jejunal lamina propria lymphocytes (n=3 piglets per group). **(H–L)** Densitometric quantification of the protein bands shown in **(G)** (n=3 piglets per group). For the data presented in this figure, a total of 24 piglets were used across the four experimental groups (0 h, 2 h, 4 h, and 8 h). Statistical significance was determined by comparing each time point to the 0 h control. For panels **(B–E)**, data normality was first assessed. For normally distributed data, an unpaired two-tailed Student’s t-test was used; Welch’s correction was applied when variances were unequal. For data that did not follow a normal distribution, the Mann–Whitney U test was performed. Welch’s t-test was applied for panels **(H–L)**. All data are expressed as mean ± SEM. ^*^P < 0.05 vs. the 0 h group.

To investigate the temporal effects of ETEC infection on jejunal lamina propria lymphocytes pyroptosis, we characterized the expression of key markers at 0, 2, 4, and 8 h post-infection. Immunofluorescence staining of the jejunal sections showed a progressive increase in CD68 fluorescence intensity and GSDMD-N expression, which reached a peak at 8 h post-infection (**Figures 2A–C**). Notably, GSDMD-N signals were partially colocalized with CD68^+^ cells (white arrows), suggesting macrophage pyroptosis. Consistently, the mRNA levels of *NLRP3*, *Caspase-1*, and *GSDMD* were significantly upregulated at 8 h post-infection compared to the 0 h control (**Figures 2D–F**). Western blot analysis demonstrated that ETEC challenge induced a time-dependent activation of the pyroptotic machinery in lamina propria lymphocytes (**Figure 2G**). Specifically, the expression of Caspase-1 and the active pore-forming fragment GSDMD-N remained relatively stable during the early stages (2–4 h) but exhibited a significant increase at 8 h post-infection (**Figures 2I, J**). Although NLRP3 protein levels showed a visible upward trend at 8 h post-infection, the difference did not reach statistical significance in this specific cell population (**Figure 2H**). Furthermore, the maturation of IL-1β, a hallmark of inflammasome activation, was markedly enhanced. The levels of Pro-IL-1β began to rise significantly at 4 h and remained elevated at 8 h (**Figure 2K**). Crucially, the mature form of the cytokine, Cleaved IL-1β (p17), showed a robust and significant increase at 8 h post-infection (**Figure 2L**). Collectively, these results indicate that ETEC infection promotes the activation of the NLRP3 inflammasome and subsequent GSDMD-mediated pyroptosis in jejunal lamina propria lymphocytes, contributing to the inflammatory response in the piglet intestine.

It is noteworthy that, after ETEC challenge in piglets, necroptosis in jejunal crypt epithelial cells was detected prior to pyroptosis in lamina propria lymphocytes, suggesting a potential role of epithelial cell necroptosis in promoting pyroptosis of lamina propria lymphocytes.

### 3.2 ETEC infection triggers temporal progression of intestinal inflammation and injury in piglets

**Figure 3:**
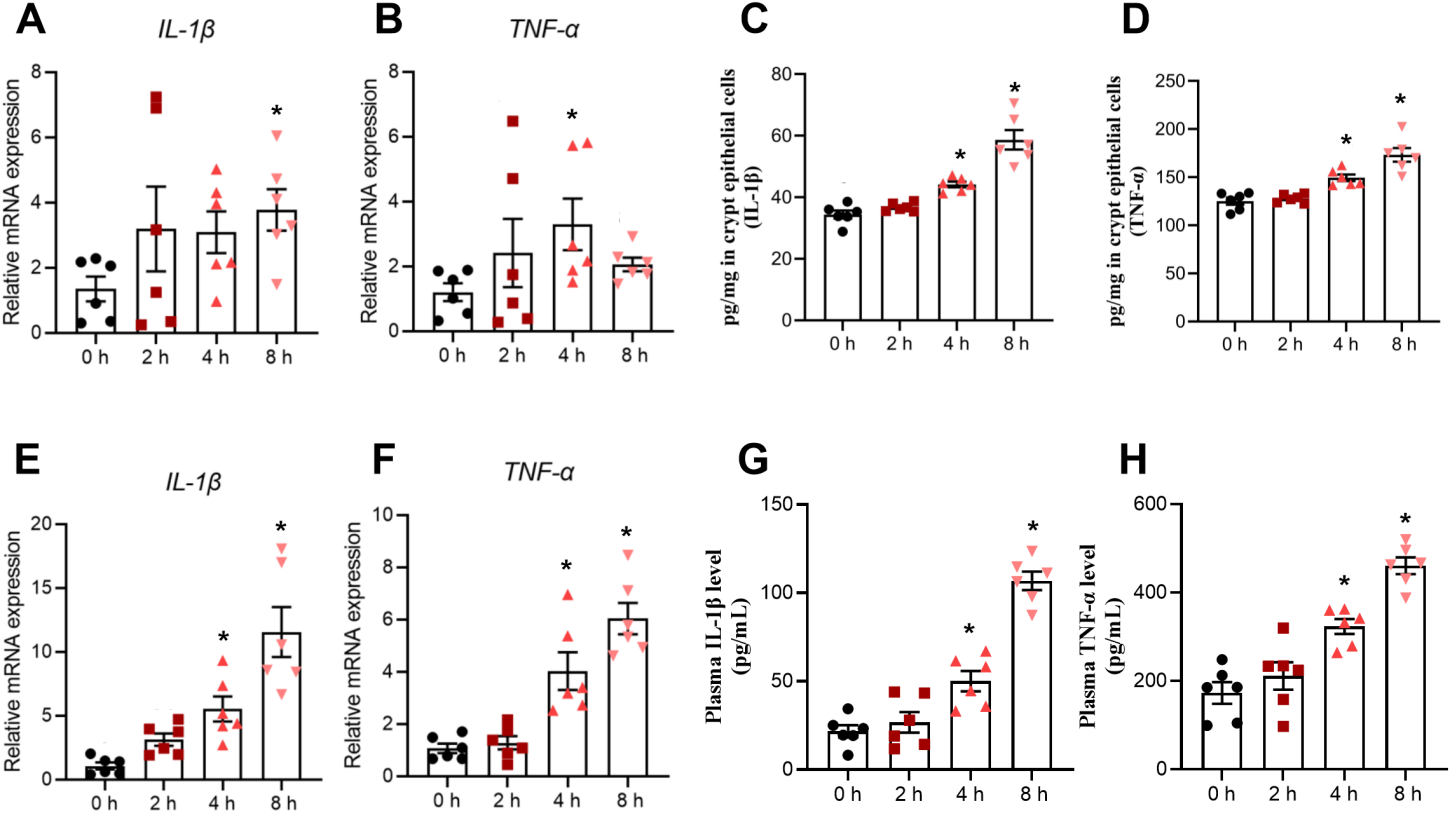
ETEC infection triggers a dynamic intestinal inflammatory response in piglets. **(A, B)** mRNA expression levels of *IL−1β* and *TNF−α* in jejunal crypt epithelial cells (n=6 piglets per group). **(C, D)** Protein expression levels of IL−1β and TNF−α in jejunal crypt epithelial cells (n=6 piglets per group). **(E, F)** mRNA expression levels of *IL−1β* and *TNF−α* in jejunal lamina propria lymphocytes (n=6 piglets per group). **(G, H)** Concentrations of plasma IL-1β and TNF-α (n=6 piglets per group). For the data presented in this figure, a total of 24 piglets were used across the four experimental groups (0 h, 2 h, 4 h, and 8 h). Each time point was compared with the 0 h control after assessing data normality. For normally distributed data, an unpaired two-tailed Student’s t-test was applied; Welch’s t-test was used when variances were unequal. For data that did not follow a normal distribution, the Mann–Whitney U test was performed. Data are presented as mean ± SEM. ^*^P < 0.05 vs. the 0 h group.

In this section, a total of 24 piglets were all subjected to ETEC challenge and allocated into four groups based on sampling time (0, 2, 4, and 8 h post-infection), conducted as one independent *in vivo* experiment. The specific number of samples (n) used for each individual assay is detailed in the corresponding figure legends. To evaluate the dynamic effects of ETEC infection on intestinal inflammation in piglets, TNF−α and IL−1β were quantified at mRNA and protein levels in jejunal crypt epithelial cells, at mRNA level in lamina propria lymphocytes, and in plasma. In jejunal crypt epithelial cells of piglets challenged with ETEC, *IL-1β* mRNA was significantly increased at 8 h (**Figure 3A**), whereas *TNF-α mRNA* was significantly elevated at 4 h (**Figure 3B**). Similarly, the protein expression of IL-1β and TNF-α in jejunal crypt epithelial cells was found to be elevated at 4–8 h post-infection (**Figures 3C, D**). The *IL-1β* and *TNF-α* mRNA levels in jejunal lamina propria lymphocytes of piglets were significantly elevated at 4–8 h post- infection (**Figures 3E, F**). In addition, significant increases in plasma IL-1β and TNF-α concentrations were observed in piglets at 4–8 h following ETEC challenge (**Figures 3G, H**). Notably, IL−1β and TNF−α levels in jejunal lamina propria lymphocytes and plasma increased sharply at 8 h post−infection compared with 4 h, likely due to pyroptosis of lamina propria lymphocytes.

**Figure 4:**
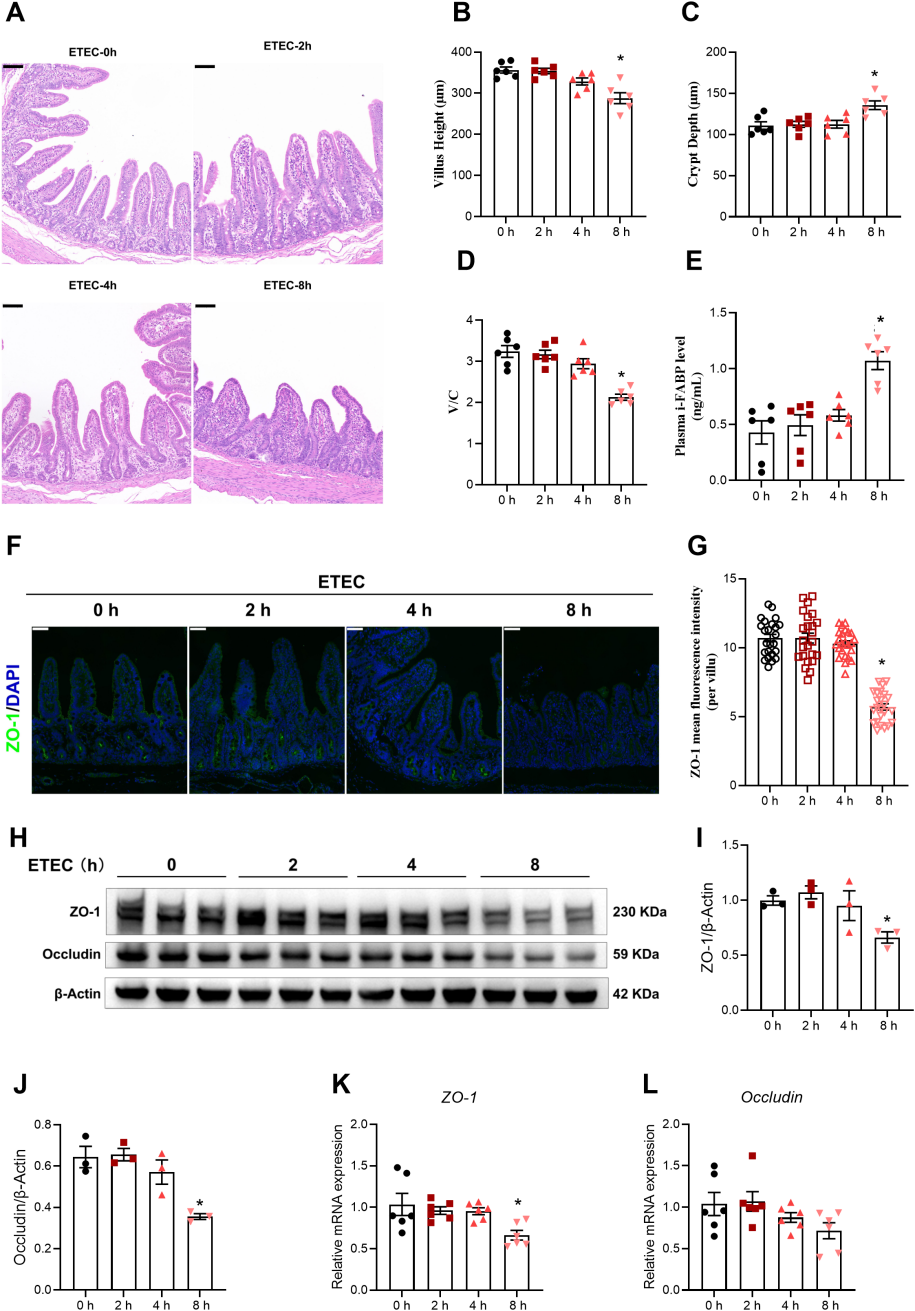
ETEC infection dynamically induces intestinal injury and barrier dysfunction in piglets. **(A)** Jejunal morphological features observed in hematoxylin and eosin−stained sections. Scale bar: 100 μm (top-left corner). **(B, C)** Jejunal villus height and crypt depth (n=6 piglets per group). **(D)** Villus height-to-crypt depth ratio of the jejunum (n=6 piglets per group). **(E)** Plasma i-FABP levels (n=6 piglets per group). **(F)** ZO-1 staining of jejunum in the indicated groups. Scale bar: 50 μm (top-left corner). **(G)** MFI of ZO-1 in in the jejunal villi. MFI quantification of ZO-1 in in the jejunal villi (n=3 piglets per group; see “Materials and Methods” for details on the quantification method). **(H–J)** Western blot analysis and densitometric quantification of ZO-1 and Occludin protein expression in the jejunal mucosa (n=3 piglets per group). **(K, L)** Relative mRNA expression of *ZO-1* and *Occludin* in the jejunal mucosa (n=6 piglets per group). For the data presented in this figure, a total of 24 piglets were used across the four experimental groups (0 h, 2 h, 4 h, and 8 h). Statistical significance was determined by comparing each time point to the 0 h control. For panels **(B–E, J–L)**, data normality was first assessed. For normally distributed data, an unpaired two-tailed Student’s t-test was used; Welch’s t-test was applied when variances were unequal. For data that did not follow a normal distribution, the Mann–Whitney U test was performed. For panels **(I-K)**, comparisons were performed using Welch’s t-test. All data are expressed as mean ± SEM. ^*^P < 0.05 vs. the 0 h group.

Subsequently, we evaluated the changes in intestinal injury−related parameters at different time points following ETEC challenge. Jejunal villus atrophy was observed at 8 h post-infection compared with the 0 h control group (**Figure 4A**). A further analysis revealed that, 8 h after ETEC challenge, jejunal villus height was reduced (**Figure 4B**), crypt depth was increased (**Figure 4C**), and the villus height-to-crypt depth ratio was decreased (**Figure 4D**). Compared with the 0 h control group, ETEC challenge elevated plasma intestinal fatty acid–binding protein (i-FABP) levels at 8 h post-infection (**Figure 4E**). Furthermore, we examined the expression of tight junction proteins, which are critical for maintaining the mucosal barrier. Immunofluorescence staining showed that the intensity of ZO-1 in the jejunal villi remained stable up to 4 h but significantly declined at 8 h post-infection (**Figures 4F, G**). This result was corroborated by Western blot analysis, which revealed a significant downregulation of ZO-1 and Occludin protein levels at the 8 h mark (**Figures 4H–J**). To further assess the impact of ETEC challenge on the transcriptional regulation of intestinal barrier components, we measured the mRNA expression levels of the tight junction proteins *ZO-1* and *Occludin* in the jejunal mucosa (**Figures 4K, L**). The results showed a time-dependent downregulation of *ZO-1* mRNA expression, which was significantly reduced at 8 h post-infection compared to the 0 h control group (**Figure 4K**). Similarly, the transcript levels of *Occludin* exhibited a progressive declining trend over time; however, these changes did not reach statistical significance at any of the observed time points (**Figure 4L**).

These findings demonstrate that ETEC challenge triggers severe intestinal barrier dysfunction by disrupting the morphological structure and degrading tight junction proteins in a time-dependent manner.

### 3.3 Verification of ETEC colonization on jejunal mucosa

In this section, a total of 10 piglets were all subjected to ETEC challenge and allocated into three groups based on sampling time (0, 4, and 8 h post-infection), conducted as one independent *in vivo* experiment. The specific number of samples (n) used for each individual assay is detailed in the corresponding figure legends. To confirm that the observed intestinal pathology was directly associated with the presence of the challenge strain, we recovered mucosal-adherent bacteria and performed genotypic verification. As shown in **Supplementary Figure 3A**, PCR analysis of the K88 (F4) fimbrial gene revealed that no K88-positive bacteria were detected in the washed jejunal mucosa of the 0 h control piglets. In contrast, all bacterial isolates recovered from the jejunal mucosa at 4 h and 8 h post-infection were K88-positive, with the expected amplicon size of 480 bp. This confirms the successful and rapid colonization of the challenge strain on the intestinal epithelia following oral inoculation. To further ensure that the recovered isolates were identical to the original challenge strain (ETEC CVCC196), PCR was performed on representative colonies from the 4 h and 8 h groups (**Supplementary Figure 3B**). The results demonstrated that these isolates possessed the complete virulence profile of the challenge strain, including the F4ac fimbriae (507 bp), O8 serogroup (208 bp), and enterotoxins STb (124 bp) and LT (274 bp). These findings provide definitive evidence that the ETEC strain successfully colonized and maintained its pathogenic potential in the jejunum throughout the 8-hour acute infection window.

### 3.4 Nec−1 suppress ETEC−induced pyroptosis of lamina propria lymphocytes by inhibiting necroptosis of crypt epithelial cells in piglets

**Figure 5:**
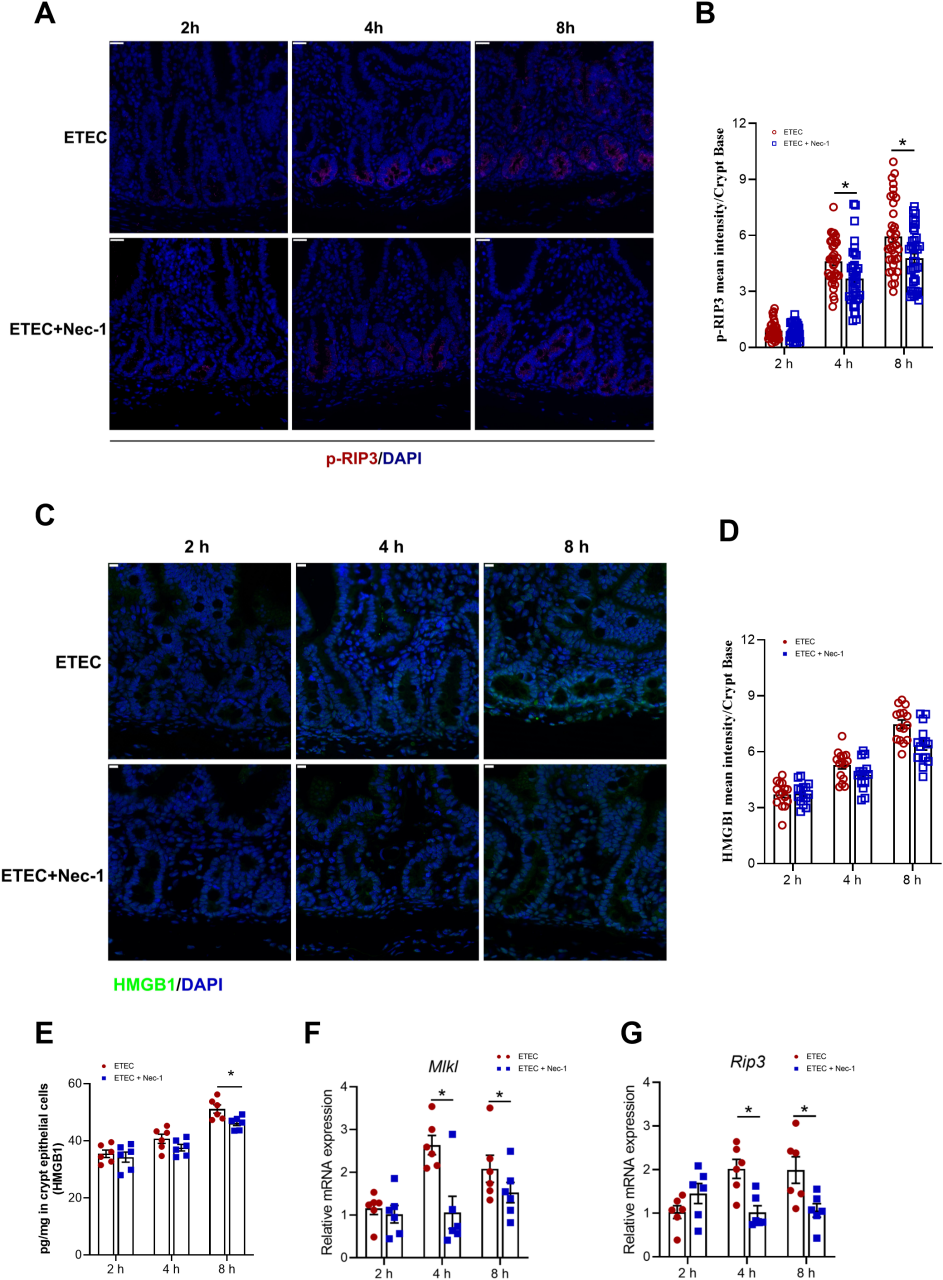
Nec-1 suppresses intestinal epithelial cell necroptosis in ETEC-challenged piglets. **(A)** p-RIP3 staining of jejunum in the indicated groups. Scale bar: 20 μm (top-left corner). **(B)** MFI of p−RIP3 in epithelial cells of the jejunal crypt region. MFI quantification of p-RIP3 at the crypt base (n=3 piglets per group; see “Materials and Methods” for details on the quantification method). **(C)** HMGB1 staining of jejunum in the indicated groups. Scale bar: 10 μm (top-left corner). **(D)** MFI of HMGB1 at the crypt base. To capture the distribution of protein expression, 15 well-defined crypts per group were randomly selected from a single biological sample (n=1 piglet per group) and analyzed as independent observation units. For specific details on the sampling and quantification procedure, see ‘Materials and Methods’. Results are shown as observed values from a single biological replicate, and no statistical tests were applied. **(E)** HMGB1 protein levels (pg/mg) in crypt epithelial cells measured by ELISA (n=6 piglets per group). **(F, G)** mRNA expression levels of *Rip3* and *MLKL* in jejunal crypt epithelial cells (n=6 piglets per group). For the data presented in this figure, a total of 36 piglets were used across 6 experimental groups (ETEC challenge at 2 h, 4 h, and 8 h, and Nec-1 pre-treatment + ETEC challenge at 2 h, 4 h, and 8 h). Comparisons between the ETEC group and the ETEC+Nec-1 group at each specific time point were performed after assessing data normality. For normally distributed data, an unpaired two-tailed Student’s t-test was used; Welch’s correction was applied when variances were unequal. For data that did not follow a normal distribution, the Mann–Whitney U test was performed. All data are expressed as mean ± SEM. ^*^P < 0.05 vs. the ETEC group at the same time point.

To evaluate the temporal effects of Nec-1 pretreatment during ETEC infection, a total of 36 piglets were utilized in this section, allocated into six experimental groups (initial n=6 piglets per group): ETEC challenge for 2 h, 4 h, and 8 h, and corresponding Nec-1 pretreatment groups (ETEC+Nec-1) at matched time points. The specific number of biological replicates analyzed for each individual assay is detailed in the respective figure legends. Nec−1 was administered 30 min prior to ETEC challenge to further verify the involvement of intestinal epithelial cell necroptosis in ETEC−induced pyroptosis of lamina propria lymphocytes. Immunofluorescence staining showed that Nec-1 treatment effectively suppressed the ETEC-induced upregulation of p-RIP3 in the jejunal crypt base at both 4 h and 8 h post-infection (**Figures 5A, B**). Consistent with the protein expression patterns, Nec-1 pretreatment significantly downregulated the ETEC-induced mRNA levels of *Mlkl* and *Rip3* at 4 h and 8 h (**Figures 5F, G**). Nec-1 pretreatment was found to lower the HMGB1 fluorescence intensity in the crypt base (**Figures 5C, D**), while significantly decreasing the HMGB1 protein levels in isolated crypt epithelial cells at the 8 h time point compared to the ETEC group (**Figure 5E**).

At 8 h post−infection, *Lyz* mRNA expression in Paneth cells was upregulated by Nec-1 pretreatment (**Supplementary Figure 2A**). Notably, Nec-1 pretreatment significantly mitigated the ETEC-induced downregulation of *Lgr5* mRNA at 4 h (**Supplementary Figure 2B**) and *Ascl2* mRNA at 8 h post−infection (**Supplementary Figure 2C**) in piglets.

**Figure 6:**
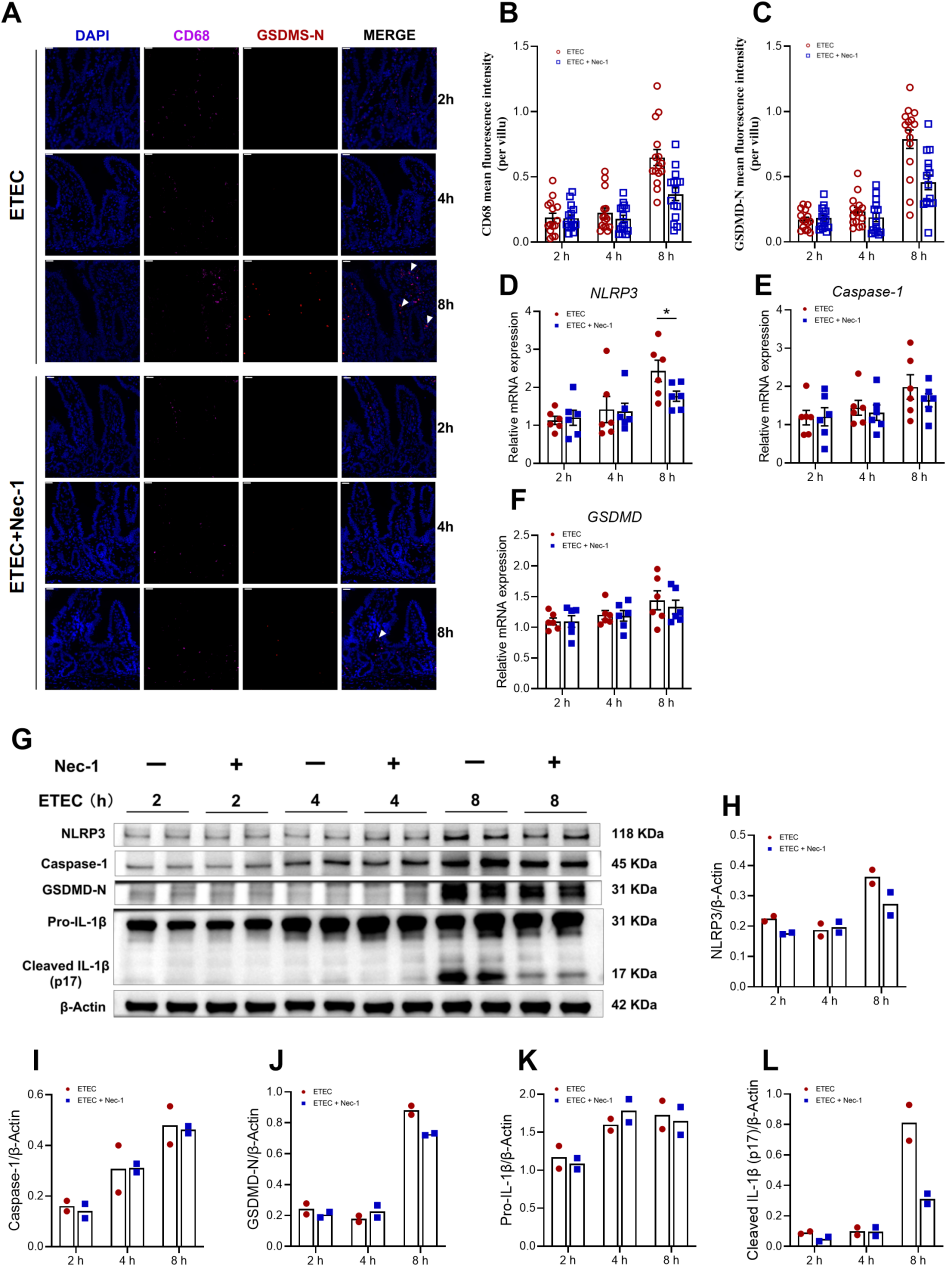
Suppression of necroptosis by Nec-1 alleviates pyroptosis triggered by ETEC in jejunal lamina propria lymphocytes of piglets. **(A)** CD68 and GSDMD-N staining of jejunum in the indicated groups. White arrows indicate the colocalization of GSDMD-N and CD68. Scale bar: 20 μm (top-left corner). **(B, C)** MFI for CD68 and GSDMD-N. To capture the distribution of protein expression, 15 well-defined villi per group were randomly selected from a single biological sample (n=1 piglet per group) and analyzed as independent observation units. For specific details on the sampling and quantification procedure, see ‘Materials and Methods’. Results are shown as observed values from a single biological replicate, and no statistical tests were applied. **(D–F)** mRNA expression levels of Caspase-1, NLRP3 and GSDMD in jejunal lamina propria lymphocytes (n=6 piglets per group). **(G)** Western blot analysis of NLRP3, Caspase-1, GSDMD-N, Pro-IL-1β, and Cleaved IL-1β (p17) in the jejunal lamina propria lymphocytes (n=2 piglets per group). **(H–L)** Densitometric quantification of the protein bands shown in **(G)** (n=2 piglets per group). For the data presented in this figure, a total of 36 piglets were used across 6 experimental groups (ETEC challenge at 2 h, 4 h, and 8 h, and Nec-1 pre-treatment + ETEC challenge at 2 h, 4 h, and 8 h). Comparisons between the ETEC and ETEC+Nec-1 groups at each specific time point were performed for panels **(B–E, G, K, L)** after assessing data normality. For normally distributed data, an unpaired two-tailed Student’s t-test was used (with Welch’s correction applied for unequal variances); for non-normally distributed data, the Mann–Whitney U test was performed. For panels **(I, J)** (n=2 piglets per group), data are presented as the mean with individual values, and no statistical comparisons were conducted. Quantitative data are expressed as mean ± SEM, except for panels **(I, J)**, which show the mean and individual values. ^*^P < 0.05 vs. the ETEC group at the same time point.

To investigate the impact of ETEC and Nec-1 on the expression of pyroptotic markers, we performed immunofluorescence staining on jejunal sections (**Figure 6A**). Notably, Nec-1 pretreatment attenuated the ETEC-induced elevation in CD68 fluorescence intensity and GSDMD-N expression at 8 h post-infection (**Figures 6B, C**). At the transcriptional level, ETEC-induced upregulation of *NLRP3* mRNA was significantly attenuated by Nec-1 at 8 h post−infection (**Figure 6D**), while *Caspase-1* and *GSDMD* mRNA levels also showed a downward trend in the Nec-1 treated group (**Figures 6E, F**). Furthermore, Western blot analysis (**Figure 6G**) confirmed that Nec-1 treatment suppressed the protein expression of NLRP3 and Caspase-1 (**Figures 6H, I**). Most importantly, the formation of the active pore-forming fragment GSDMD-N and the maturation of Cleaved IL-1β (p17) were markedly inhibited by Nec-1 at 8 h post-infection (**Figures 6J–L**).

### 3.5 Nec-1 attenuates intestinal inflammation and injury induced by ETEC challenge in piglets

**Figure 7:**
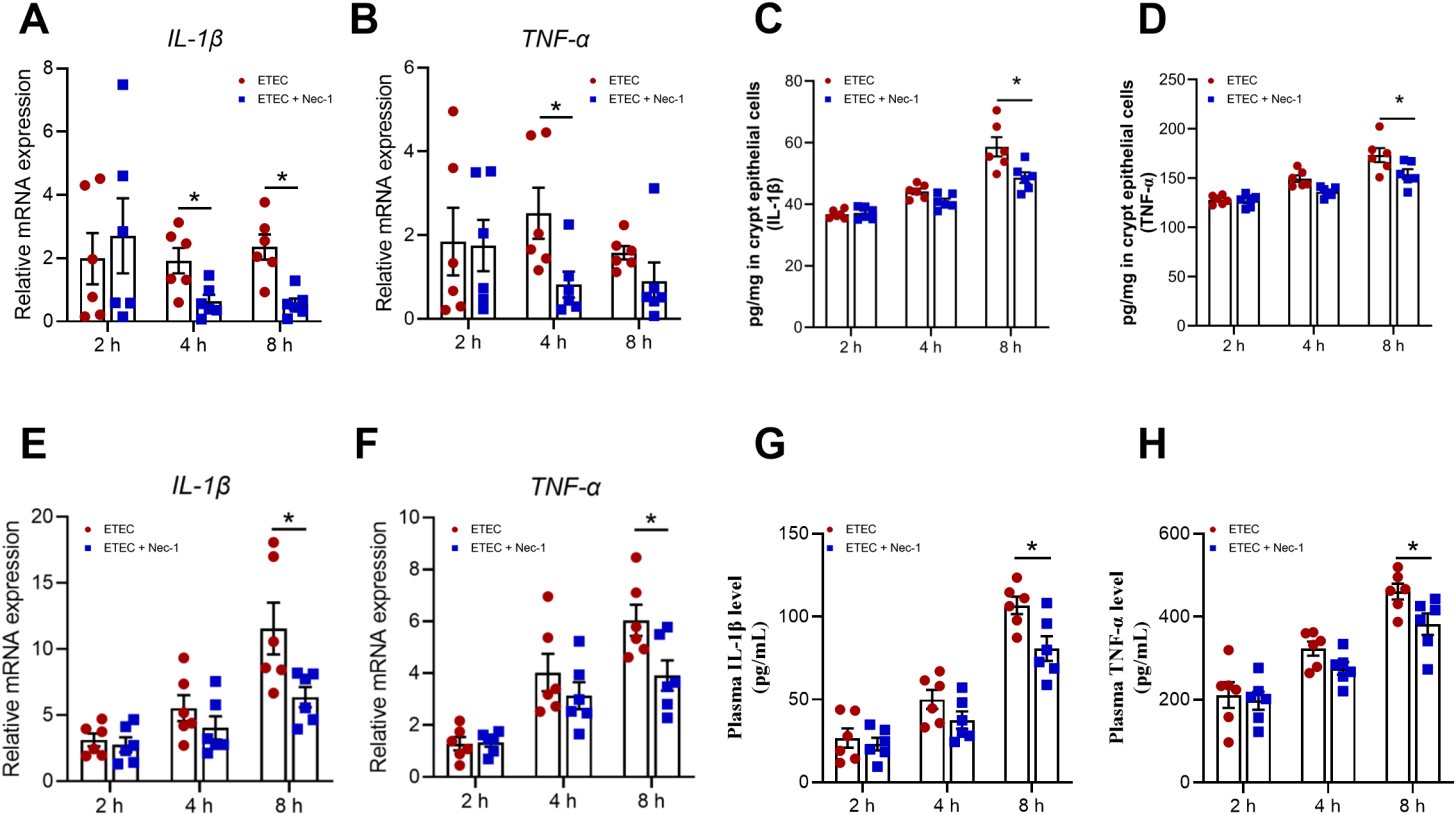
Nec-1 inhibition of necroptosis attenuated ETEC-induced intestinal inflammation in piglets. **(A, B)** mRNA expression levels of IL−1β and TNF−α in jejunal crypt epithelial cells (n=6 piglets per group). **(C, D)** Protein expression levels of IL−1β and TNF−α in jejunal crypt epithelial cells (n=6 piglets per group). **(E, F)** mRNA expression levels of IL−1β and TNF−α in jejunal lamina propria lymphocytes (n=6 piglets per group). **(G, H)** Concentrations of plasma IL-1β and TNF-α (n=6 piglets per group). For the data presented in this figure, a total of 36 piglets were used across 6 experimental groups (ETEC challenge at 2 h, 4 h, and 8 h, and Nec-1 pre-treatment + ETEC challenge at 2 h, 4 h, and 8 h). Comparisons between the ETEC group and the ETEC+Nec-1 group at each specific time point were performed after assessing data normality. For normally distributed data, an unpaired two-tailed Student’s t-test was applied; Welch’s t-test was used when variances were unequal. For data that did not follow a normal distribution, the Mann–Whitney U test was performed. Data are presented as mean ± SEM. ^*^P < 0.05 vs. the ETEC group at the same time point.

To evaluate the temporal effects of Nec-1 pretreatment during ETEC infection, a total of 36 piglets were utilized in this section, allocated into six experimental groups (initial n=6 piglets per group): ETEC challenge for 2 h, 4 h, and 8 h, and corresponding Nec-1 pretreatment groups (ETEC+Nec-1) at matched time points. The specific number of biological replicates analyzed for each individual assay is detailed in the respective figure legends. The impact of Nec-1–mediated inhibition of necroptosis on intestinal inflammation in piglets following ETEC challenge was examined. Nec-1 pretreatment significantly suppressed the ETEC-induced elevations of *IL-1β* mRNA (**Figure 7A**) in jejunal crypt epithelial cells at 4 h and 8 h, and *TNF-α* mRNA (**Figure 7B**) at 4 h, compared with the control group. Furthermore, at 8 h after ETEC challenge, Nec-1 pretreatment significantly suppressed the elevations in IL-1β (**Figure 7C**) and TNF-α (**Figure 7D**) protein levels in jejunal crypt epithelial cells compared with the control group. At 8 h post-infection, Nec-1 pretreatment significantly suppressed ETEC-induced increases in *IL-1β* (**Figure 7E**) and *TNF-α* (**Figure 7F**) mRNA expression in jejunal lamina propria lymphocytes, compared with the control group. Compared with controls, Nec-1 pretreatment significantly suppressed the ETEC-induced upregulation of plasma IL-1β (**Figure 7G**) and TNF-α (**Figure 7H**) protein levels at 8 h post-infection.

**Figure 8:**
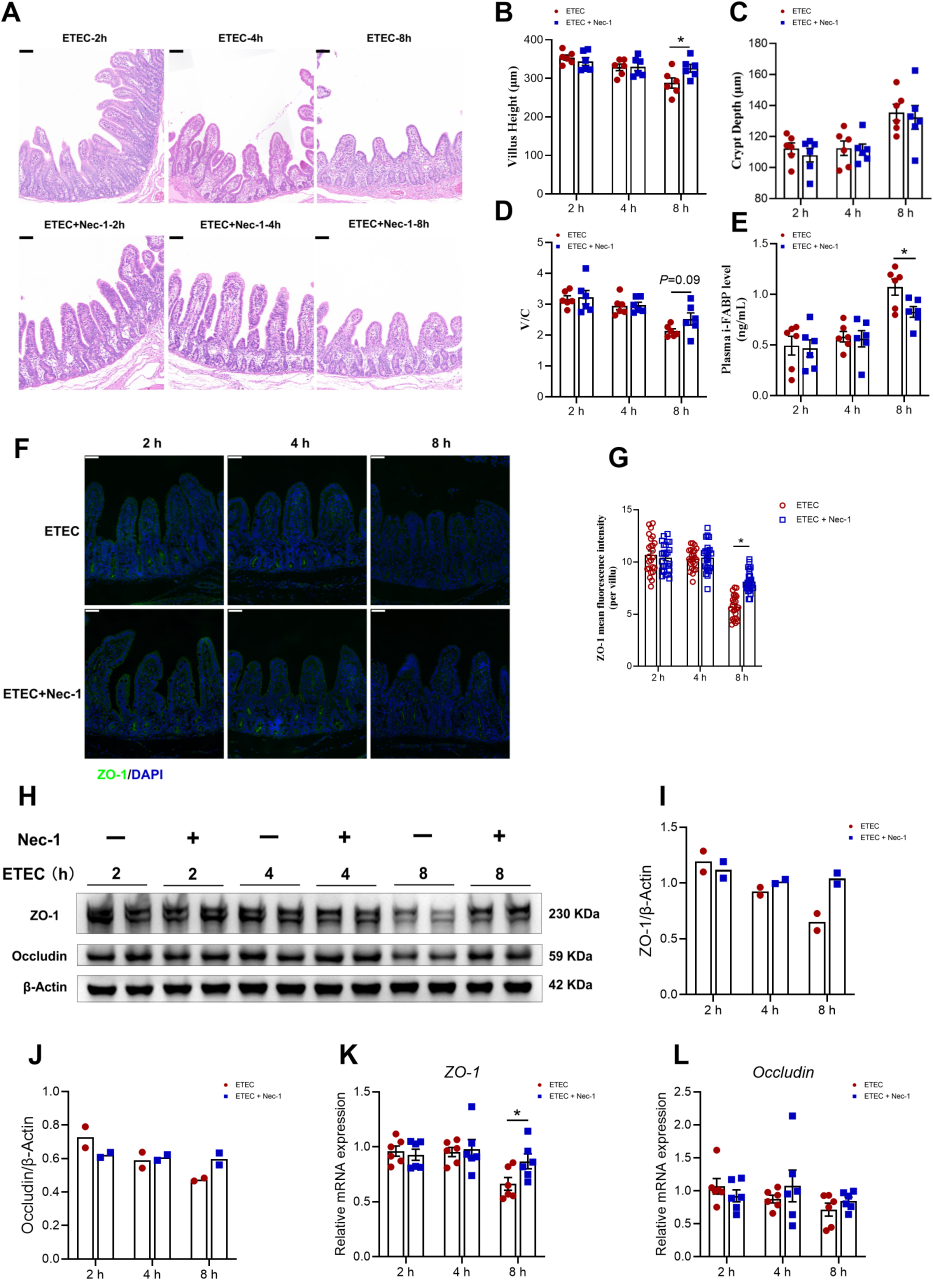
Nec-1 attenuates intestinal injury and barrier dysfunction induced by ETEC challenge in piglets. **(A)** Jejunal morphological features observed in hematoxylin and eosin−stained sections. Scale bar: 100 μm (top-left corner). **(B, C)** Jejunal villus height and crypt depth (n=6 piglets per group). **(D)** Villus height-to-crypt depth ratio of the jejunum (n=6 piglets per group). **(E)** Plasma i-FABP levels (n=6 piglets per group). **(F)** ZO-1 staining of jejunum in the indicated groups. Scale bar: 50 μm (top-left corner). **(G)** MFI of ZO-1 in in the jejunal villi. MFI quantification of ZO-1 in in the jejunal villi (n=3 piglets per group; see “Materials and Methods” for details on the quantification method). **(H–J)** Western blot analysis and densitometric quantification of ZO-1 and Occludin protein expression in the jejunal mucosa (n=2 piglets per group). **(K, L)** Relative mRNA expression of *ZO-1* and *Occludin* in the jejunal mucosa (n=6 piglets per group). For the data presented in this figure, a total of 36 piglets were used across 6 experimental groups (ETEC challenge at 2 h, 4 h, and 8 h, and Nec-1 pre-treatment + ETEC challenge at 2 h, 4 h, and 8 h). For panels **(B–E, G, K, L)**, comparisons between the ETEC group and the ETEC+Nec-1 group at each specific time point were performed after assessment of data normality. For normally distributed data, an unpaired two-tailed Student’s t-test was used (with Welch’s correction applied for unequal variances); for non-normally distributed data, the Mann–Whitney U test was performed. For panels **(I, J)** (n=2 piglets per group), data are presented as the mean with individual values shown, and no statistical analyses were conducted. Data are expressed as mean ± SEM unless otherwise indicated. ^*^P < 0.05 vs. the ETEC group at the same time point.

Finally, we investigated whether Nec-1 pretreatment could alleviate intestinal injury induced by ETEC challenge in piglets (**Figure 8A**). The ETEC-induced reduction in jejunal villus height (**Figure 8B**) at 8 h post-infection was effectively alleviated by Nec-1 pretreatment in piglets, whereas crypt depth (**Figure 8C**) and the villus height-to-crypt depth ratio (**Figure 8D**) were not significantly affected. Nec-1–mediated inhibition of necroptosis attenuated the ETEC-induced elevation of plasma i-FABP levels in piglets at 8 h post-infection (**Figure 8E**). Specifically, immunofluorescence analysis revealed that Nec-1 significantly maintained the mean fluorescence intensity of ZO-1 in the villi at 8 h post-infection (**Figures 6F, G**). Consistently, the mRNA expression of ZO-1 was significantly higher in the Nec-1 group at 8 h compared to the ETEC group (**Figure 6K**). Although statistical analysis was not performed for Western blot data due to the limited number of biological replicates, Nec-1 pretreatment appeared to increase the protein levels of ZO-1 and Occludin at 8 h post-infection (**Figures 6H–J**).

## 4 Discussion

The combination of immature intestinal development and early weaning stress renders piglets vulnerable to environmental pathogens, particularly enterotoxigenic ETEC. This vulnerability results in intestinal inflammation, epithelial damage, and diarrhea, accompanied by multiple inflammatory forms of programmed cell death (17, 24). However, previous studies have not provided *in vivo* evidence for that ETEC infection in piglets induces necroptosis in small intestinal epithelial cells, nor have they elucidated the relationship between epithelial necroptosis and pyroptosis in lamina propria lymphocytes (18, 25). By using timed sampling of ETEC-challenged piglets and necroptosis inhibitor, our study first demonstrated that ETEC infection in piglets induces necroptosis of epithelial cells in the jejunal crypts *in vivo*. Moreover, we showed that ETEC-induced necroptosis of epithelial cells triggers pyroptosis in lamina propria macrophages. This sequential programmed cell death plays a key role in ETEC-induced intestinal inflammation. Therefore, our results indicated that targeting upstream epithelial cell necroptosis represents an important strategy for the attenuation of inflammation and the preservation of barrier integrity.

In the present *in vivo* study, notable intestinal epithelial damage and barrier dysfunction were observed in piglets just 8 hours post-challenge with ETEC CVCC196. While 8 hours represents an acute phase of infection, this timeframe was deliberately selected and aligns with established *in vivo* models designed to capture primary host-pathogen interactions. In highly susceptible piglets, ETEC pathogenesis is exceptionally rapid. Studies utilizing oral challenge models frequently assess the mucosa at 6 to 12 hours post-infection to investigate acute enterotoxin delivery, massive fluid hypersecretion, and primary barrier disruption, thereby avoiding the confounding secondary tissue damage caused by severe systemic dehydration and ischemia typical of later clinical stages (24–48 hours) (25, 26). The early epithelial alterations observed are biologically consistent with ETEC dynamics. Research indicates that the robust adhesion of F4^+^ ETEC to the enterocyte brush border can initiate rapid host cell signaling, leading to localized cytoskeletal reorganization, alterations in microvillar architecture, and disruption of tight junction integrity (e.g., ZO-1 and occludin redistribution) within hours of colonization (27, 28).

In the present study, a high inoculum dose (10^10–^10^11^ CFU) of ETEC CVCC196 was employed, creating a ‘super-infection’ model to characterize acute host-pathogen interactions within a condensed 8-hour window. Our microbiological analysis of washed jejunal mucosa confirmed that this dose leads to rapid and massive colonization as early as 4 h post-infection (**Supplementary Figure 3A**). Compared to standard challenge doses (typically10^8^ CFU), this magnitude likely accelerates colonization kinetics by rapidly saturating host F4 receptors. Consequently, the overwhelming presence of PAMPs, such as LPS and flagellin, may trigger a more robust and synchronized inflammatory signaling cascade through TLR4 and TLR5 (29). Furthermore, the high-affinity binding of F4ac fimbriae to specific host receptors, such as MUC4, triggers downstream signaling pathways that can lead to cytoskeletal rearrangements and disruption of the brush border membrane integrity, facilitating the subsequent action of enterotoxins (27, 30). The high concentration of enterotoxins (LT/STb) produced during ETEC colonization likely accelerates cell-death pathways (e.g., apoptosis) before compensatory host mechanisms can engage. STb in particular has been demonstrated to induce in intestinal epithelial cells (31). Additionally, many classic porcine O8 ETEC strains produce α-hemolysin (HlyA), a pore-forming toxin that may induce rapid cytotoxicity and membrane permeabilization in host epithelial cells during early infection (32). This model thus serves as a rigorous challenge to the intestinal barrier, providing clear insights into the primary mechanisms of ETEC-induced mucosal damage.”

Intestinal injury and barrier dysfunction frequently trigger intestinal inflammation (33, 34). In this study, ETEC infection induced inflammation in both small intestinal epithelial cells and lamina propria lymphocytes of piglets, with epithelial inflammation preceding that of lamina propria lymphocytes. Consistent with our results, Liu et al. (25) reported significant increases in jejunal mucosal *TNF-α* mRNA and serum TNF-α levels 3 h post-infection in piglets. Furthermore, research has demonstrated that plasma IL-1β and TNF-α levels in control piglets were markedly elevated at 9  h post-infection relative to baseline (0 h) (35). Consistent with these reports, the present study observed elevated levels of TNF-α and IL-1β in the jejunal crypt epithelial cells, lamina propria lymphocytes, and plasma of piglets following ETEC challenge. It is noteworthy that at 8 h post-infection, TNF-α mRNA expression in crypt epithelial cells showed no significant increase compared to the 0 h control, whereas its protein levels remained markedly elevated. This temporal dissociation may be attributed to the rapid degradation of TNF-α mRNA following its synthesis at this specific time point (36). As a classic early-response gene, TNF-α mRNA is characterized by high instability and rapid turnover, which likely results in a transient transcriptional burst followed by a localized resetting, even as the translated protein continues to accumulate or persist within the cellular compartment.

A substantial body of evidence indicates that HMGB1 levels in intestinal tissues are markedly elevated following intestinal injury (37–40). In this study, we observed markedly elevated HMGB1 expression levels in jejunal crypt epithelial cells at 4 h post-ETEC challenge. Notably, the increase in HMGB1 protein levels in crypt epithelial cells following ETEC challenge occurred concurrently with the upregulation of p-RIP3, supporting the notion that HMGB1 elevation is associated with necroptosis. HMGB1 binds TLR4 and subsequently activates the MyD88–NF-κB signaling cascade. Activated NF-κB enhances the transcription of inflammatory genes, including NLRP3, pro-IL-1β, and pro-IL-18, thereby providing the “first signal” (Signal 1) required for pyroptosis initiation (16, 41). HMGB1 can also activate the NF-κB signaling cascade through its interaction with RAGE (42), thereby contributing to downstream inflammatory responses and facilitating inflammasome priming. In the present study, HMGB1 elevation in jejunal crypt epithelial cells occurred earlier than the onset of pyroptosis in lamina propria lymphocytes. Notably, ETEC infection-induced intestinal barrier dysfunction in piglets leads to the translocation of PAMPs (such as LPS) from the intestinal lumen into the lamina propria. LPS also acts as the first signal (priming signal) to up-regulate the expression of NLRP3 inflammasome components and pro-inflammatory cytokine precursors via the TLR4/NF-κB pathway, thereby establishing the molecular basis for macrophage pyroptosis (43). In addition, the current study showed that ETEC infection induced epithelial inflammation prior to that of lamina propria lymphocytes. Furthermore, the necroptotic rupture of epithelial cells triggers the release of ATP. This extracellular ATP activates P2X7R to induce K^+^ efflux, which provides the critical activation signal (Signal 2) for the assembly of the NEK7-NLRP3-ASC-Caspase-1 complex and the subsequent execution of pyroptosis (44). Consequently, HMGB1 and ATP released from necroptotic jejunal epithelial cells following ETEC challenge is likely to promote pyroptosis in lamina propria lymphocytes.

In the course of macrophage pyroptosis, pro-inflammatory cytokines including IL-1β and IL-18 undergo marked maturation and are abundantly secreted, concomitant with the release of diverse intracellular DAMPs. This amplifies the activation of neighboring immune cells and precipitates an intense cytokine storm, culminating in tissue injury, barrier dysfunction, and systemic inflammation (17, 45). In this experiment, we observed that 8 h following ETEC challenge, lymphocytes within the jejunal lamina propria showed markedly elevated expression of pyroptosis-associated proteins and genes. Immunofluorescence staining further confirmed abundant pyroptotic cells (GSDMD-N–positive) in the lamina propria, a subset of which were identified as macrophages. Plasma IL-1β levels were substantially higher at 8 h post-ETEC challenge than at 4 h, and pyroptosis of lymphocytes within the jejunal lamina propria was likely among the factors that contributed to this increase. While prior studies showed that ETEC infection induces pyroptosis in piglet small-intestinal mucosal cells (18, 25), the role of this form of cell death in driving ETEC-associated intestinal inflammation remains uncharacterized. Our findings support that ETEC-provoked pyroptosis of lamina propria lymphocytes represents a primary driver of the amplification and advancement of intestinal inflammatory processes.

Previous studies have demonstrated that RIP3 protein is highly expressed in crypt Paneth cells, which are susceptible to necroptosis upon exposure to inflammatory stimuli (46–48). Crypt epithelial cells are located deep within the intestinal glands, in close proximity to lamina propria macrophages and lymphocytes. Necroptotic cell death in crypt cells may facilitate the rapid release of DAMPs, which could efficiently reach nearby immune cells and potentially contribute to the activation of pyroptotic pathways. In the current study, we showed that ETEC induced necroptosis mainly occurred in the crypts, which are primarily composed of Paneth cells and stem cells. Notably, the pathogenesis of ETEC-induced intestinal injury appears to involve a complex interplay of multiple cell death pathways, aligning with the emerging concept of PANoptosis. While previous studies have reported apoptosis in the intestinal epithelium of ETEC-infected piglets (49), our findings specifically identified necroptosis within crypt epithelial cells. Furthermore, although it has been reported that ETEC triggers pyroptosis in the IPEC-1 cell line *in vitro (*9), our *in vivo* immunofluorescence data revealed that pyroptotic signaling was primarily concentrated in the lamina propria rather than the epithelium (**Figure 2A**). Nevertheless, the overlapping presence of these various cell death markers suggests that ETEC infection may promote PANoptosome assembly within porcine small intestinal epithelial cells. Consequently, our current data do not allow us to definitively determine whether the observed phenotype reflects ‘pure’ necroptosis or serves as a component of a broader PANoptotic response—a distinction that warrants further experimental validation. Paneth cells and intestinal stem cell function are important for maintaining mucosal integrity during ETEC infection in piglets. Paneth cells secrete antimicrobial peptides (e.g., defensins) and lysozyme that constrain ETEC colonization and modulate the crypt microbiota, while providing niche signals (Wnt, epidermal growth factor, and Notch ligands) that sustain LGR5^+^ stem-cell function (50). In agreement with our *in vivo* observations, *in vitro* experiments have demonstrated that heat-stable enterotoxin (STp) produced by ETEC inhibits Lgr5 expression in porcine intestinal organoids by suppressing Wnt/β-catenin signaling (51). Intestinal stem cells drive rapid epithelial turnover and injury repair, thereby restoring barrier integrity after toxin-mediated damage (52). It is noteworthy that previous research showed that direct exposure of murine macrophages (J774A.1 cells) to ETEC triggers pyroptosis via activation of the NLRP3 inflammasome pathway (53). Therefore, it cannot be excluded that ETEC-induced barrier dysfunction allows luminal bacteria to translocate into the lamina propria and directly infect resident macrophages, which may further contribute to the induction of macrophage pyroptosis in the intestinal mucosa. While Nec-1 inhibits the necroptosis-pyroptosis axis, its role in strengthening barrier integrity cannot be overlooked. By minimizing epithelial cell loss and subsequent bacterial translocation into the lamina propria, Nec-1 may indirectly reduce the inflammatory stimuli that drive lamina propria lymphocytes pyroptosis. Moreover, ETEC-induced necroptosis of epithelial cells is not only a key factor in inducing macrophage pyroptosis, but also may impair the repair function of intestinal epithelium by damaging the functions of Paneth cells and stem cells, thus further aggravating the infection. Accordingly, in the current study, we found that pretreatment with Nec-1 alleviates ETEC-mediated injury to Paneth cells and intestinal stem cells, thereby limiting intraluminal ETEC expansion and maintenance of tight junctions in intestinal epithelial cells.

Consistent with our *in vivo* observations, Xiao et al. (9) reported that ETEC induces necroptosis in the porcine small intestinal epithelial cell line (IPEC-1) through activation of the RIP3–MLKL pathway. However, the mechanism underlying ETEC infection–induced necroptosis in porcine small intestinal epithelial cells remains to be elucidated. According to Yu et al. (54), *Escherichia coli*–derived LPS induces epithelial cell necroptosis during sepsis via upregulation of RIP1–RIP3–MLKL pathway-related proteins. Moreover, we hypothesize that ETEC infection results in elevated TNF-α levels in the lamina propria, and that TNF-α can promote classical TNF-induced necroptosis via TNFR1 expressed on intestinal epithelial cells. Notably, oral administration of the heat-stable enterotoxin from ETEC in mice can induce intestinal inflammation and injury. Therefore, the possibility cannot be excluded that ETEC-derived enterotoxins directly induce necroptosis in porcine small intestinal epithelial cells (51). Future research will aim to further elucidate the underlying mechanisms through which ETEC infection induces necroptosis of intestinal Paneth cells in piglets.Although our experimental design included comprehensive time-course evaluations (a 0 h baseline control, ETEC-challenged groups at 2, 4, and 8 h, and Nec-1 pre-treated + ETEC groups at corresponding time points), we acknowledge a limitation in the current study: the absence of a ‘Nec-1 alone’ control group. Including piglets treated solely with Nec-1 would have provided valuable baseline physiological information. Specifically, it would have clarified whether pharmacological inhibition of RIP1 by Nec-1 independently alters basal intestinal barrier function, tight junction protein turnover, or baseline inflammatory cytokine profiles in healthy, uninfected piglets. Furthermore, a ‘Nec-1 alone’ group would help conclusively rule out any potential off-target effects, mild cytotoxicity, or compensatory mechanisms triggered by the inhibitor itself on normal porcine enterocytes. While previous studies have extensively established the safety and specificity of Nec-1 in various models, future investigations incorporating a vehicle-treated Nec-1 control are warranted to fully decouple the drug’s inherent effects on intestinal homeostasis from its protective efficacy against ETEC-induced necroptosis. Despite the significant protective effects observed, several limitations of this study should be acknowledged. Primarily, the sample size used for the *in vivo* experiments (*n*=6 piglets per group) is relatively limited, largely due to strict ethical considerations regarding large animal welfare and the logistical complexities of *in vivo* challenge trials. Consequently, inter-individual variability—a common and unavoidable characteristic in outbred animal models like piglets—must be addressed as a potential confounding factor. While this inherent biological variation might contribute to data fluctuation, the consistent statistical differences observed across multiple parameters (e.g., tight junction proteins, cytokines, and necroptosis markers) strongly support the reliability of our overall conclusions. Nevertheless, future studies utilizing larger sample sizes will be beneficial to minimize the impact of individual differences.

## 5 Conclusion

**Figure 9:**
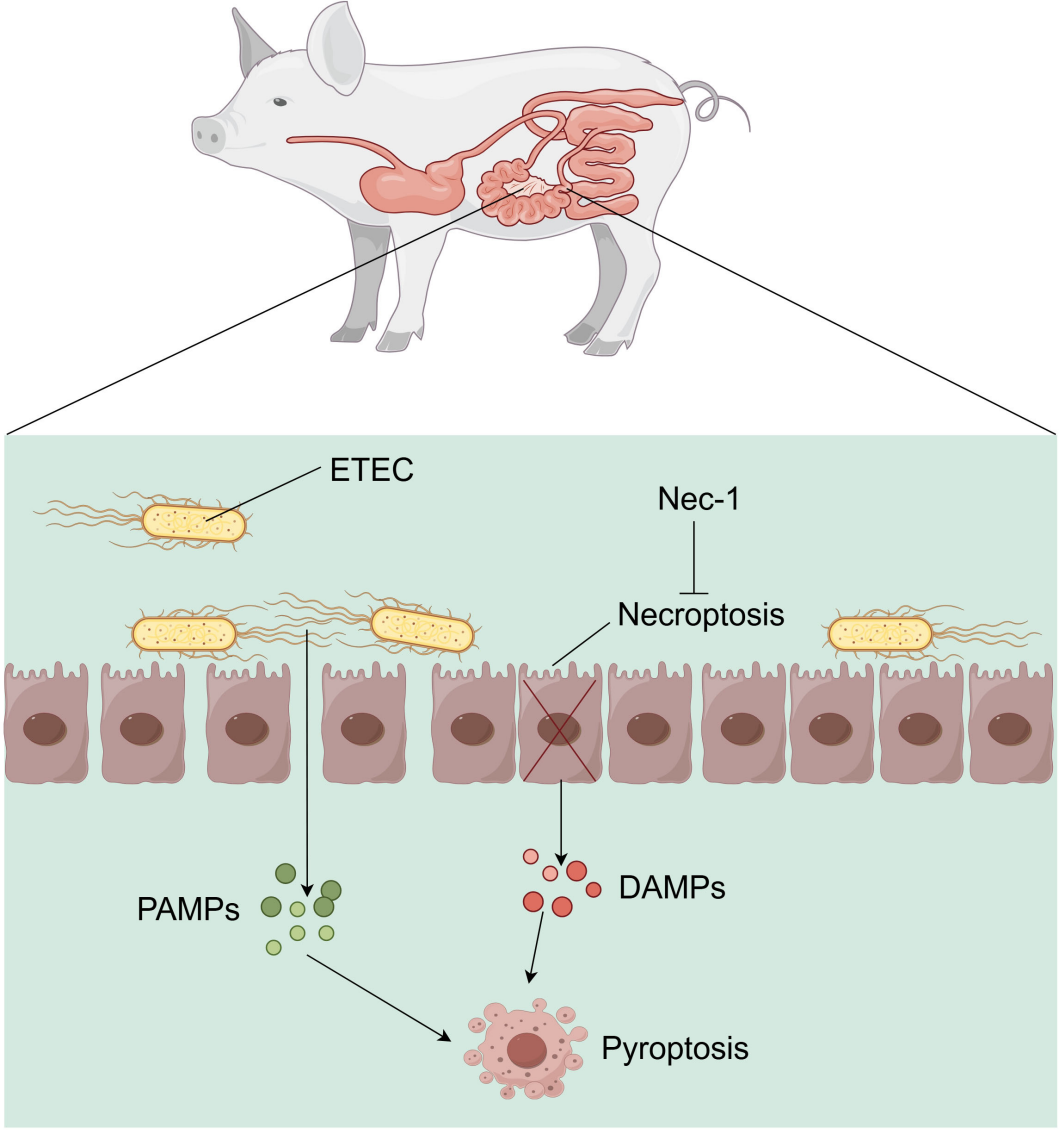
Mechanism of ETEC-induced necroptosis in small intestinal epithelial cells promoting immune cell pyroptosis in the lamina propria of piglets.

In conclusion, our findings indicate that ETEC infection in piglets induces necroptosis of crypt epithelial cells, with the released HMGB1 potentially exacerbating intestinal inflammation and injury by promoting pyroptosis of lamina propria lymphocytes (**Figure 9**).

## Data Availability

The original contributions presented in the study are included in the article/**Supplementary Material**. Further inquiries can be directed to the corresponding author.

## Supplementary material

Supplementary Figure 1 ETEC infection induces dynamic changes in the expression of marker genes of jejunal Paneth cells and stem cells in piglets. **(A–C)** mRNA expression levels of Lyz, Lgr5 and Ascl2 in jejunal crypt epithelial cells (n=6 piglets per group). For the data presented in this figure, a total of 24 piglets were used across the four experimental groups (0 h, 2 h, 4 h, and 8 h). Each time point was compared with the 0 h control after assessing data normality. For normally distributed data, an unpaired two-tailed Student’s t-test was applied; Welch’s t-test was used when variances were unequal. For data that did not follow a normal distribution, the Mann–Whitney U test was performed. Data are presented as mean ± SEM. ^*^*P* < 0.05 vs. the 0 h group.

Supplementary Figure 2 Nec−1 mitigates intestinal stem cell damage in piglets challenged with ETEC. **(A–C)** mRNA expression levels of Lyz, Lgr5 and Ascl2 in jejunal crypt epithelial cells (n=6 piglets per group). For the data presented in this figure, a total of 36 piglets were used across 6 experimental groups (ETEC challenge at 2 h, 4 h, and 8 h, and Nec-1 pre-treatment + ETEC challenge at 2 h, 4 h, and 8 h). Comparisons between the ETEC group and the ETEC+Nec-1 group at each specific time point were performed after assessing data normality. For normally distributed data, an unpaired two-tailed Student’s t-test was applied; Welch’s t-test was used when variances were unequal. For data that did not follow a normal distribution, the Mann–Whitney U test was performed. Data are presented as mean ± SEM. ^*^*P* < 0.05 vs. the ETEC group at the same time point.

Supplementary Figure 3 Genotypic verification of ETEC colonization on the jejunal mucosa of piglets. A PCR amplification of the ETEC K88 fimbrial gene (480 bp) from mucosal-adherent bacteria recovered from the jejunum of piglets at 0 (n=2 piglets per group), 4 (n=4 piglets per group), and 8 h (n=4 piglets per group) post-infection. The presence of the K88 gene confirms the successful colonization of the challenge strain. Lane M: DNA molecular weight marker. B PCR detection of specific virulence factors in representative bacterial isolates recovered from the jejunal mucosa at 4 h (n=1 piglet per group) and 8 h (n=1 piglet per group) post-infection. Amplified genes include F4ac (507 bp), O8 (208 bp), enterotoxin STb (124 bp), enterotoxin LT (274 bp), and K88 (480 bp), confirming that the colonized bacteria maintained the complete virulence profile of the challenge strain ETEC CVCC196. Lane M: DNA molecular weight marker. For the data presented in this figure, a total of 10 piglets were used across the thre experimental groups (0 h, 4 h, and 8 h).

## References

[1] Sun Y Kim SW . Intestinal challenge with enterotoxigenic Escherichia coli in pigs, and nutritional intervention to prevent postweaning diarrhea. Anim Nutr. (2017) 3:322–30. doi: 10.1016/j.aninu.2017.10.001. PMID: 29767133 PMC5941267

[2] Amezcua R Friendship RM Dewey CE Gyles C Fairbrother JM . Presentation of postweaning Escherichia coli diarrhea in southern Ontario, prevalence of hemolytic E. coli serogroups involved, and their antimicrobial resistance patterns. Can J Vet Res. (2002) 66:73. 11989737 PMC226986

[3] Wang L Zhu Y Zhang L Guo L Wang X Pan Z . Mechanisms of PANoptosis and relevant small-molecule compounds for fighting diseases. Cell Death Dis. (2023) 14:851. doi: 10.1038/s41419-023-06370-2. PMID: 38129399 PMC10739961

[4] Bedoui S Herold MJ Strasser A . Emerging connectivity of programmed cell death pathways and its physiological implications. Nat Rev Mol Cell Biol. (2020) 21:678–95. doi: 10.1038/s41580-020-0270-8. PMID: 32873928

[5] Liu T Zong H Chen X Li S Liu Z Cui X . Toll-like receptor 4-mediated necroptosis in the development of necrotizing enterocolitis. Pediatr Res. (2022) 91:73–82. doi: 10.1038/s41390-021-01457-y. PMID: 33731807 PMC8770135

[6] Siegmund D Ehrenschwender M Wajant H . TNFR2 unlocks a RIPK1 kinase activity-dependent mode of proinflammatory TNFR1 signaling. Cell Death Dis. (2018) 9:921. doi: 10.1038/s41419-018-0973-3. PMID: 30206205 PMC6134143

[7] Hu S Chang X Zhu H Wang D Chen G . PI3K mediates tumor necrosis factor induced-necroptosis through initiating RIP1-RIP3-MLKL signaling pathway activation. Cytokine. (2020) 129:155046. doi: 10.1016/j.cyto.2020.155046. PMID: 32114297

[8] Liccardi G Annibaldi A . MLKL post-translational modifications: road signs to infection, inflammation and unknown destinations. Cell Death Differ. (2023) 30:269–78. doi: 10.1038/s41418-022-01061-5. PMID: 36175538 PMC9520111

[9] Xiao K Zhou M Lv Q He P Qin X Wang D . Protocatechuic acid and quercetin attenuate ETEC-caused IPEC-1 cell inflammation and injury associated with inhibition of necroptosis and pyroptosis signaling pathways. J Anim Sci Biotechnol. (2023) 14:5. doi: 10.1186/s40104-022-00816-x. PMID: 36721159 PMC9890695

[10] Xiao K Yang Y Zhang Y Lv Q Huang F Wang D . Long chain PUFA ameliorate ETEC-induced intestinal inflammation and cell injury by modulating pyroptosis and necroptosis signaling pathways in IPEC-1 cells. Br J Nutr. (2022) 128:1–36. doi: 10.1017/S0007114521005213. PMID: 35470787

[11] Fu J Wu H . Structural mechanisms of NLRP3 inflammasome assembly and activation. Annu Rev Immunol. (2023) 41:301–16. doi: 10.1146/annurev-immunol-081022-021207. PMID: 36750315 PMC10159982

[12] Blevins HM Xu Y Biby S Zhang S . The NLRP3 inflammasome pathway: a review of mechanisms and inhibitors for the treatment of inflammatory diseases. Front Aging Neurosci. (2022) 14:879021. doi: 10.3389/fnagi.2022.879021. PMID: 35754962 PMC9226403

[13] Lechuga S Ivanov AI . Disruption of the epithelial barrier during intestinal inflammation: Quest for new molecules and mechanisms. Biochim Biophys Acta (BBA) Mol Cell Res. (2017) 1864:1183–94. doi: 10.1016/j.bbamcr.2017.03.007. PMID: 28322932 PMC5507344

[14] Macura B Kiecka A Szczepanik M . Intestinal permeability disturbances: Causes, diseases and therapy. Clin Exp Med. (2024) 24:232. doi: 10.1007/s10238-024-01496-9. PMID: 39340718 PMC11438725

[15] Kojima T Shindo Y Konno T Kodera Y Arai W Miyakawa M . Dysfunction of epithelial permeability barrier induced by HMGB1 in 2.5 D cultures of human epithelial cells. Tissue Barriers. (2022) 10:1972760. doi: 10.1080/21688370.2021.1972760. PMID: 34538217 PMC9067465

[16] Shang L Wang L Shi X Wang N Zhao L Wang J . HMGB1 was negatively regulated by HSF1 and mediated the TLR4/MyD88/NF-κB signal pathway in asthma. Life Sci. (2020) 241:117120. doi: 10.1016/j.lfs.2019.117120. PMID: 31825792

[17] Fu J Jiang Z Wen C Su W Yang M He H . Bacterial infection in weaned piglets promotes diarrhea by inducing the NLRP3 inflammasome-pyroptosis pathway. Sci China Life Sci. (2025) 68:1–16. doi: 10.1007/s11427-024-2728-2. PMID: 40622657

[18] Li HH Li YP Zhu Q Qiao JY Wang WJ . Dietary supplementation with Clostridium butyricum helps to improve the intestinal barrier function of weaned piglets challenged with enterotoxigenic Escherichia coli K88. J Appl Microbiol. (2018) 125:964–75. doi: 10.1111/jam.13936. PMID: 29851202

[19] Xia F . Toxicity Test and Therapeutic Effect of Diweng Keli on Piglet white Scour (thesis/dissertation). Jilin University (2015).

[20] Essa MOA Basher NS Abdelhadi LAM Ibrahim NA Rehman SU Husien HM . Robust goat-derived enterococcus isolates with broad-spectrum antipathogenic activity as next-generation probiotic candidates. Vet Sci. (2026) 13:120. doi: 10.3390/vetsci13020120. PMID: 41745914 PMC12945012

[21] Liu Y Xu Q Wang Y Liang T Li X Wang D . Necroptosis is active and contributes to intestinal injury in a piglet model with lipopolysaccharide challenge. Cell Death Dis. (2021) 12:62. doi: 10.1038/s41419-020-03365-1. PMID: 33431831 PMC7801412

[22] Livak KJ Schmittgen TD . Analysis of relative gene expression data using real-time quantitative PCR and the 2- ΔΔCT method. Methods. (2001) 25:402–8. doi: 10.1006/meth.2001.1262. PMID: 11846609

[23] Schmittgen TD Livak KJ . Analyzing real-time PCR data by the comparative CT method. Nat Protoc. (2008) 3:1101–8. doi: 10.1038/nprot.2008.73. PMID: 18546601

[24] Kim SW Duarte ME . Understanding intestinal health in nursery pigs and the relevant nutritional strategies. Anim Biosci. (2021) 34:338. doi: 10.5713/ab.21.0010. PMID: 33705620 PMC7961202

[25] Li H Liu X Shang Z Qiao J . Clostridium butyricum helps to alleviate inflammation in weaned piglets challenged with enterotoxigenic Escherichia coli K88. Front Vet Sci. (2021) 8:683863. doi: 10.3389/fvets.2021.683863. PMID: 34277756 PMC8282889

[26] Middelkoop A Kettunen H Guan X Vuorenmaa J Tichelaar R Gambino M . Effect of dietary tall oil fatty acids and hydrolysed yeast in SNP2-positive and SNP2-negative piglets challenged with F4 enterotoxigenic Escherichia coli. Sci Rep. (2024) 14:2060. doi: 10.1038/s41598-024-52586-3. PMID: 38267615 PMC10808182

[27] Xia P Song Y Zou Y Yang Y Zhu G . F4+ enterotoxigenic Escherichia coli (ETEC) adhesion mediated by the major fimbrial subunit FaeG. J Basic Microbiol. (2015) 55:1118–24. doi: 10.1002/jobm.201400901. PMID: 25847483

[28] Roselli M Finamore A Hynönen U Palva A Mengheri E . Differential protection by cell wall components of Lactobacillus amylovorus DSM 16698Tagainst alterations of membrane barrier and NF-kB activation induced by enterotoxigenic F4+ Escherichia coli on intestinal cells. BMC Microbiol. (2016) 16:226. doi: 10.1186/s12866-016-0847-8. PMID: 27688074 PMC5041403

[29] Saha S Namai F Nishiyama K Villena J Kitazawa H . Role of immunomodulatory probiotics in alleviating bacterial diarrhea in piglets: a systematic review. J Anim Sci Biotechnol. (2024) 15:112. doi: 10.1186/s40104-024-01070-z. PMID: 39129013 PMC11318305

[30] Schroyen M Stinckens A Verhelst R Niewold T Buys N . The search for the gene mutations underlying enterotoxigenic Escherichia coli F4ab/ac susceptibility in pigs: a review. Vet Res. (2012) 43:70. doi: 10.1186/1297-9716-43-70. PMID: 23061722 PMC3499147

[31] Butt S Saleh M Gagnon J . Impact of the Escherichia coli heat-stable enterotoxin b (STb) on gut health and function. Toxins (Basel). (2020) 12:760. doi: 10.3390/toxins12120760. PMID: 33276476 PMC7761119

[32] Burgos Y Beutin L . Common origin of plasmid encoded alpha-hemolysin genes in Escherichia coli. BMC Microbiol. (2010) 10:193. doi: 10.1186/1471-2180-10-193. PMID: 20637130 PMC2918590

[33] Shin W Kim HJ . Intestinal barrier dysfunction orchestrates the onset of inflammatory host–microbiome cross-talk in a human gut inflammation-on-a-chip. Proc Natl Acad Sci. (2018) 115:E10539–47. doi: 10.1073/pnas.1810819115. PMID: 30348765 PMC6233106

[34] Lechuga S Braga-Neto MB Naydenov NG Rieder F Ivanov AI . Understanding disruption of the gut barrier during inflammation: Should we abandon traditional epithelial cell lines and switch to intestinal organoids? Front Immunol. (2023) 14:1108289. doi: 10.3389/fimmu.2023.1108289. PMID: 36875103 PMC9983034

[35] Ren C Wang Y Lin X Song H Zhou Q Xu W . A combination of formic acid and monolaurin attenuates enterotoxigenic Escherichia coli induced intestinal inflammation in piglets by inhibiting the NF-κB/MAPK pathways with modulation of gut microbiota. J Agric Food Chem. (2020) 68:4155–65. doi: 10.1021/acs.jafc.0c01414. PMID: 32202779

[36] Kontoyiannis D Pasparakis M Pizarro TT Cominelli F Kollias G . Impaired on/off regulation of TNF biosynthesis in mice lacking TNF AU-rich elements: implications for joint and gut-associated immunopathologies. Immunity. (1999) 10:387–96. doi: 10.1016/S1074-7613(00)80038-2. PMID: 10204494

[37] Chen X Fang D Li L Chen L Li Q Gong F . Glycyrrhizin ameliorates experimental colitis through attenuating interleukin-17-producing T cell responses via regulating antigen-presenting cells. Immunol Res. (2017) 65:666–80. doi: 10.1007/s12026-017-8894-2. PMID: 28108937

[38] Török S Almási N Valkusz Z Pósa A Varga C Kupai K . Investigation of H2S donor treatment on neutrophil extracellular traps in experimental colitis. Int J Mol Sci. (2021) 22:12729. doi: 10.3390/ijms222312729. PMID: 34884536 PMC8657984

[39] Yamasaki H Mitsuyama K Masuda J Kuwaki K Takedatsu H Sugiyama G . Roles of high-mobility group box 1 in murine experimental colitis. Mol Med Rep. (2009) 2:23–7. doi: 10.3892/mmr_00000056, PMID: 21475785

[40] Chen Y-Z Li C Gu J Lv S Song J Tang Z . Anti-oxidative and immuno-protective effect of camel milk on radiation-induced intestinal injury in C57BL/6 J mice. Dose-Response. (2021) 19:15593258211003798. doi: 10.1177/15593258211003798. PMID: 33867894 PMC8020251

[41] Singh H Agrawal DK . Therapeutic potential of targeting the HMGB1/RAGE axis in inflammatory diseases. Molecules. (2022) 27:7311. doi: 10.3390/molecules27217311. PMID: 36364135 PMC9658169

[42] Qin Y-H Dai S-M Tang G-S Zhang J Ren D Wang Z-W . HMGB1 enhances the proinflammatory activity of lipopolysaccharide by promoting the phosphorylation of MAPK p38 through receptor for advanced glycation end products. J Immunol. (2009) 183:6244–52. doi: 10.4049/jimmunol.0900390. PMID: 19890065

[43] Zhang W-J Li K-Y Lan Y Zeng H-Y Chen S-Q Wang H . NLRP3 Inflammasome: A key contributor to the inflammation formation. Food Chem Toxicol. (2023) 174:113683. doi: 10.1016/j.fct.2023.113683. PMID: 36809826

[44] Di Virgilio F Dal Ben D Sarti AC Giuliani AL Falzoni S . The P2X7 receptor in infection and inflammation. Immunity. (2017) 47:15–31. doi: 10.1016/j.immuni.2017.06.020. PMID: 28723547

[45] Karki R Kanneganti T-D . The ‘cytokine storm’: molecular mechanisms and therapeutic prospects. Trends Immunol. (2021) 42:681–705. doi: 10.1016/j.it.2021.06.001. PMID: 34217595 PMC9310545

[46] Günther C Martini E Wittkopf N Amann K Weigmann B Neumann H . Caspase-8 regulates TNF-α-induced epithelial necroptosis and terminal ileitis. Nature. (2011) 477:335–9. 10.1038/nature10400 PMC3373730 21921917

[47] Cui C Wang F Zheng Y Wei H Peng J . From birth to death: The hardworking life of Paneth cell in the small intestine. Front Immunol. (2023) 14:1122258. doi: 10.3389/fimmu.2023.1122258. PMID: 36969191 PMC10036411

[48] Lueschow SR McElroy SJ . The Paneth cell: the curator and defender of the immature small intestine. Front Immunol. (2020) 11:587. doi: 10.3389/fimmu.2020.00587. PMID: 32308658 PMC7145889

[49] Xia Y Bin P Liu S Chen S Yin J Liu G . Enterotoxigenic Escherichia coli infection promotes apoptosis in piglets. Microb Pathogen. (2018) 125:290–4. doi: 10.1016/j.micpath.2018.09.032. PMID: 30243552

[50] Cray P Sheahan BJ Dekaney CM . Secretory sorcery: paneth cell control of intestinal repair and homeostasis. Cell Mol Gastroenterol Hepatol. (2021) 12:1239–50. doi: 10.1016/j.jcmgh.2021.06.006. PMID: 34153524 PMC8446800

[51] Zhou J Huang D Gao C Yan H Zou S Wang X . Heat-stable enterotoxin inhibits intestinal stem cell expansion to disrupt the intestinal integrity by downregulating the Wnt/β-catenin pathway. Stem Cells. (2021) 39:482–96. doi: 10.1002/stem.3324. PMID: 33373490

[52] Santos AJ Lo Y-H Mah AT Kuo CJ . The intestinal stem cell niche: homeostasis and adaptations. Trends Cell Biol. (2018) 28:1062–78. doi: 10.1016/j.tcb.2018.08.001. PMID: 30195922 PMC6338454

[53] Cheng Y Xiao X Fu J Zong X Lu Z Wang Y . Escherichia coli K88 activates NLRP3 inflammasome-mediated pyroptosis *in vitro* and *in vivo*. Biochem Biophys Rep. (2024) 38:101665. doi: 10.1016/j.bbrep.2024.101665. PMID: 38419757 PMC10900769

[54] Yu X Yuan J Shi L Dai S Yue L Yan M . Necroptosis in bacterial infections. Front Immunol. (2024) 15:1394857. doi: 10.3389/fimmu.2024.1394857. PMID: 38933265 PMC11199740

