# Supplementary figures and images for: Enterotoxigenic Escherichia coli–induced intestinal epithelial necroptosis drives lamina propria immune cell pyroptosis and mucosal injury in piglets
Source: Front Immunol. 2026 Mar 19;17:1778258. doi: 10.3389/fimmu.2026.1778258 (PMC13043375; doi:10.3389/fimmu.2026.1778258)

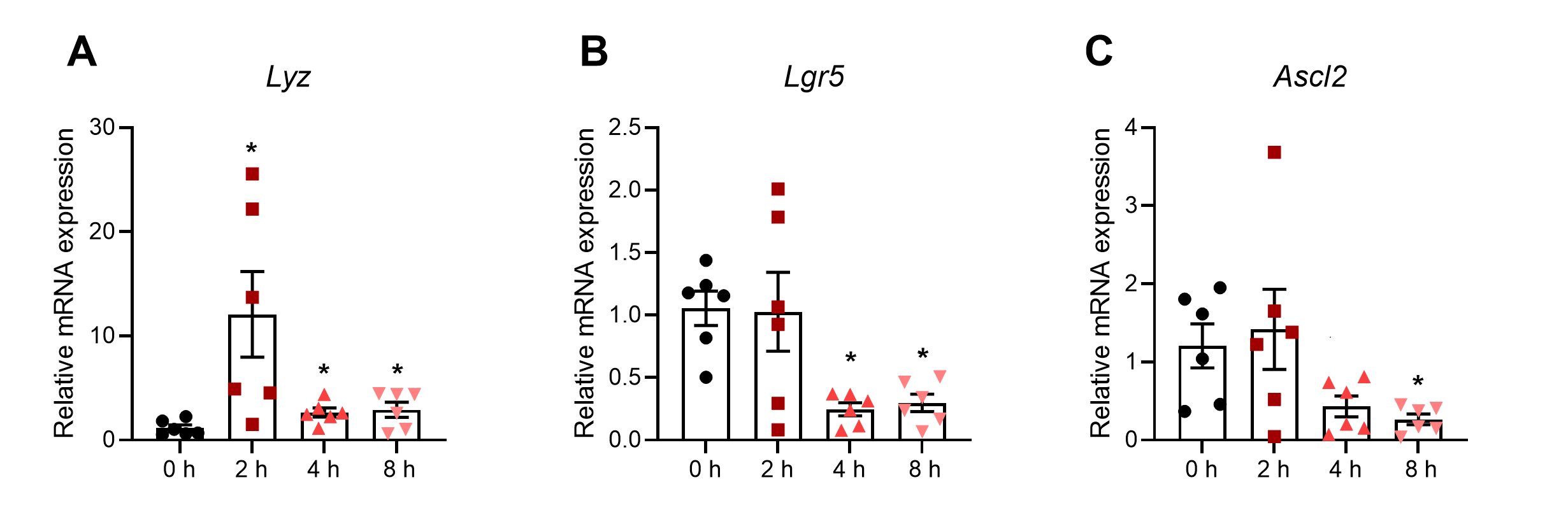

Supplement: Supplementary Figure 1 — ETEC infection induces dynamic changes in the expression of marker genes of jejunal Paneth cells and stem cells in piglets. (A–C) mRNA expression levels of Lyz, Lgr5 and Ascl2 in jejunal crypt epithelial cells (n=6 piglets per group). For the data presented in this figure, a total of 24 piglets were used across the four experimental groups (0 h, 2 h, 4 h, and 8 h). Each time point was compared with the 0 h control after assessing data normality. For normally distributed data, an unpaired two-tailed Student’s t-test was applied; Welch’s t-test was used when variances were unequal. For data that did not follow a normal distribution, the Mann–Whitney U test was performed. Data are presented as mean ± SEM. *P < 0.05 vs. the 0 h group. [file Image1.tif]

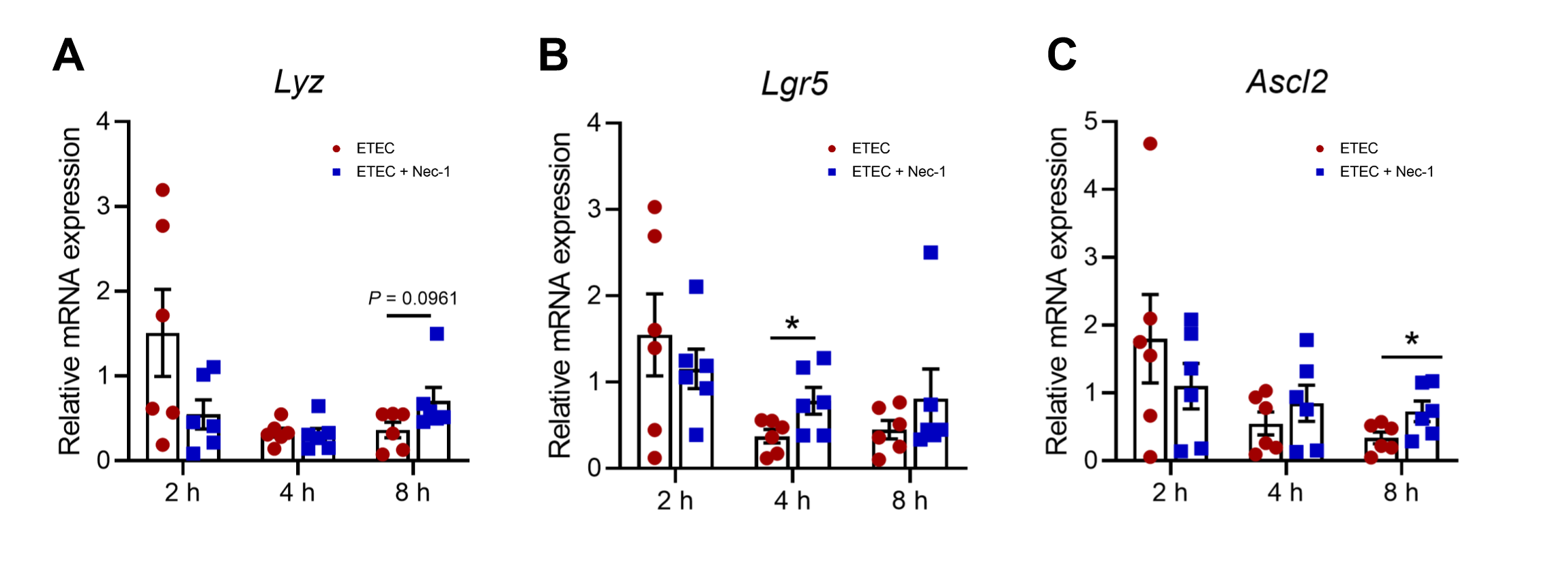

Supplement: Supplementary Figure 2 — Nec−1 mitigates intestinal stem cell damage in piglets challenged with ETEC. (A–C) mRNA expression levels of Lyz, Lgr5 and Ascl2 in jejunal crypt epithelial cells (n=6 piglets per group). For the data presented in this figure, a total of 36 piglets were used across 6 experimental groups (ETEC challenge at 2 h, 4 h, and 8 h, and Nec-1 pre-treatment + ETEC challenge at 2 h, 4 h, and 8 h). Comparisons between the ETEC group and the ETEC+Nec-1 group at each specific time point were performed after assessing data normality. For normally distributed data, an unpaired two-tailed Student’s t-test was applied; Welch’s t-test was used when variances were unequal. For data that did not follow a normal distribution, the Mann–Whitney U test was performed. Data are presented as mean ± SEM. *P < 0.05 vs. the ETEC group at the same time point. [file Image2.tif]

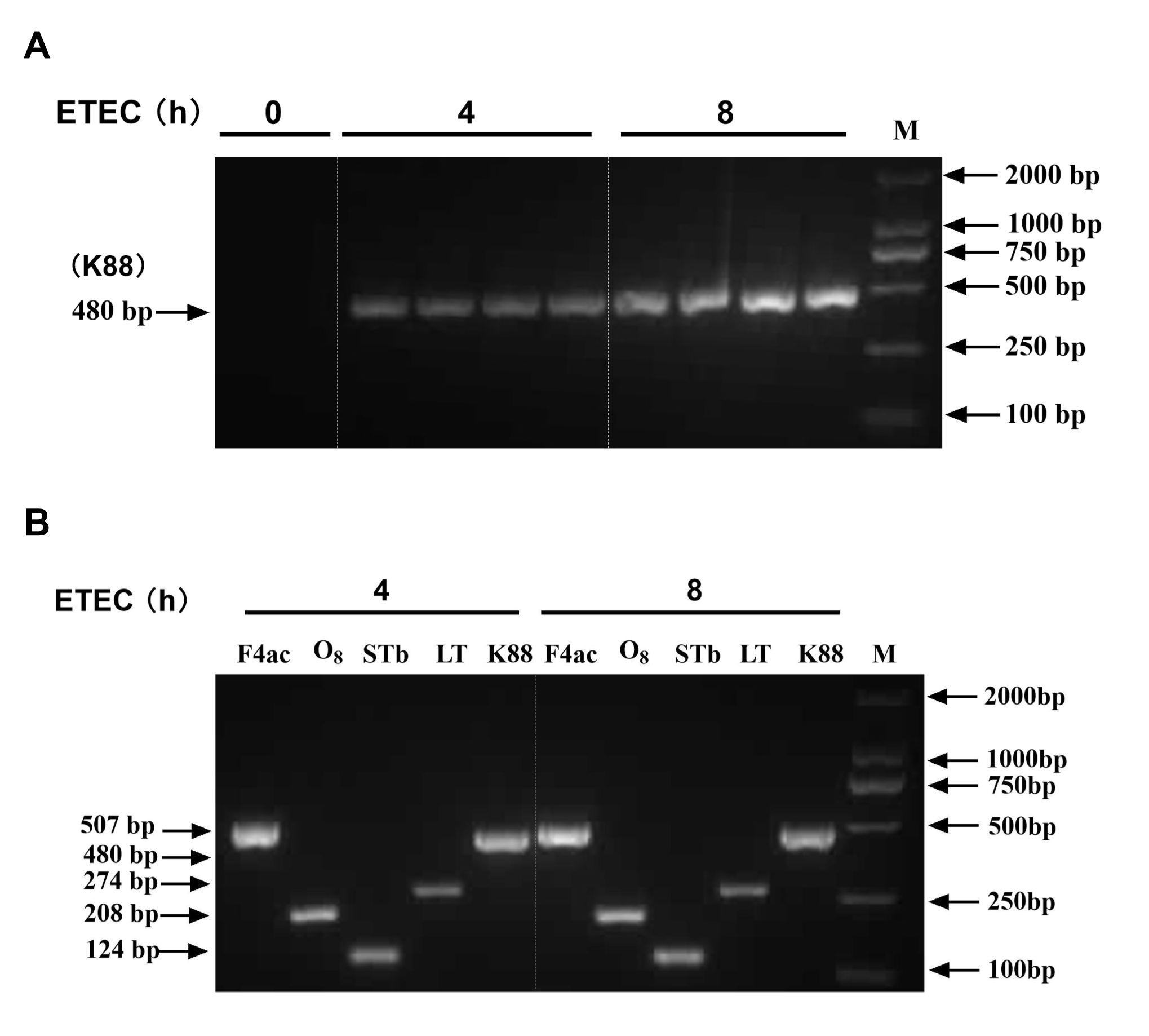

Supplement: Supplementary Figure 3 — Genotypic verification of ETEC colonization on the jejunal mucosa of piglets. A PCR amplification of the ETEC K88 fimbrial gene (480 bp) from mucosal-adherent bacteria recovered from the jejunum of piglets at 0 (n=2 piglets per group), 4 (n=4 piglets per group), and 8 h (n=4 piglets per group) post-infection. The presence of the K88 gene confirms the successful colonization of the challenge strain. Lane M: DNA molecular weight marker. B PCR detection of specific virulence factors in representative bacterial isolates recovered from the jejunal mucosa at 4 h (n=1 piglet per group) and 8 h (n=1 piglet per group) post-infection. Amplified genes include F4ac (507 bp), O8 (208 bp), enterotoxin STb (124 bp), enterotoxin LT (274 bp), and K88 (480 bp), confirming that the colonized bacteria maintained the complete virulence profile of the challenge strain ETEC CVCC196. Lane M: DNA molecular weight marker. For the data presented in this figure, a total of 10 piglets were used across the thre experimental groups (0 h, 4 h, and 8 h). [file Image3.tif]
