# Supplementary material for: Enterotoxigenic Escherichia coli–induced intestinal epithelial necroptosis drives lamina propria immune cell pyroptosis and mucosal injury in piglets
Source: Front Immunol. 2026 Mar 19;17:1778258. doi: 10.3389/fimmu.2026.1778258 (PMC13043375; doi:10.3389/fimmu.2026.1778258)
Supplement: Supplementary file 4 [file Table1.docx]

**Supplementary Table 1**

Primer sequences for qPCR.

| **Gene** | **Forward primer 5′-3**′ | **Reverse primer 5′-3′** |
| --- | --- | --- |
| NLRP3 | AGCAGATTCCAGTGCATCAAAG | CCTGGTGAAGCGTTTGTTGAG |
| Caspase-1 | GAGCTGAAGGCATTTGCTGCCC | CCACGGCAAGCCTGGATAATG |
| GSDMD | TGTTCGTGGTGACGGAGGTG | TGAAGGTTCGCTGCTTCTTGTC |
| MLKL | TCGCATGAGTGTGTCCAGTC | TTGGTGGCTTGAGGCTACAG |
| Rip3 | CTTGTTGTCTGTCCGTGAGC | GAGGAGGTTGGGCTGTTGA |
| ZO-1 | ATCTCGGAAAAGTGCCAGGA | CCTTCCCCTCAGAAACCCAT |
| Occludin | CTACTCGTCCAACGGGAAAG | ACGCCTCCAAGTTACCACTG |
| TNF- α | CCACCAACGTTTTCCTCACT | TAGTCGGGCAGGTTGATCTC |
| IL-1β | GCTAACTACGGTGACAACAATAATG | CTTCTCCACTGCCACGATGA |
| Lyz | GGTCTATGATCGGTGCGAGT | AACTGCTTTGGGTGTCTTGC |
| Lgr5 | CCTTGGCCCTGAACAAAATA | ATTTCTTTCCCAGGGAGTGG |
| Ascl2 | GAGCTGCTCGACTTCTCCAG | TTCCACACTAGCCCTTGGTC |
| GAPDH | ACATCATCCCTGCTTCTACTGG | CTCGGACGCCTGCTTCAC |
| F4ac | GGTGATTTCAATGGTTCGGTC | CCCAGCCGACGATTCAGAACCCCT |
| O_8_ | CAGGCACGCTATCAAACTAG | CGCTCTGACCTTTATCCAGCA |
| STb | GCTACAAATGCCTATGCATCTACACA | CATGCTCCAGCAGTACCATCTCTAAC |
| LT | ACGGCGTTACTATCCTGTCTATGTGC | TTGGTCTCGGTCAGATATGTGATTCT |
| K88 | TGAATGACCTGACCAATGGTGGAACC | GCGTTTACTCTTTGAATCTGTCCGAG |
